# A Robust Observation, Planning, and Control Pipeline for Autonomous Rendezvous with Tumbling Targets

**DOI:** 10.3389/frobt.2021.641338

**Published:** 2021-09-17

**Authors:** Keenan Albee, Charles Oestreich, Caroline Specht, Antonio Terán Espinoza, Jessica Todd, Ian Hokaj, Roberto Lampariello, Richard Linares

**Affiliations:** ^1^ Space Systems Laboratory (SSL) and Astrodynamics, Space Robotics and Controls Lab (ARCLab), Department of Aeronautics and Astronautics, Massachusetts Institute of Technology, Cambridge, MA, United States; ^2^ Guidance & Control Group, The Charles Stark Draper Laboratory, Inc., Cambridge, MA, United States; ^3^ Autonomy and Teleoperation, Institute of Robotics and Mechatronics, German Aerospace Center (DLR), Oberpfaffenhofen, Germany; ^4^ Human Systems Laboratory (HSL), Department of Aeronautics and Astronautics and Department of Applied Ocean Physics and Engineering, Massachusetts Institute of Technology/Woods Hole Oceanographic Institute, Cambridge, MA, United States

**Keywords:** space robotics, motion planning, robust tube MPC, visual estimation, on-orbit servicing, planning under uncertainty

## Abstract

Accumulating space debris edges the space domain ever closer to cascading Kessler syndrome, a chain reaction of debris generation that could dramatically inhibit the practical use of space. Meanwhile, a growing number of retired satellites, particularly in higher orbits like geostationary orbit, remain nearly functional except for minor but critical malfunctions or fuel depletion. Servicing these ailing satellites and cleaning up “high-value” space debris remains a formidable challenge, but active interception of these targets with autonomous repair and deorbit spacecraft is inching closer toward reality as shown through a variety of rendezvous demonstration missions. However, some practical challenges are still unsolved and undemonstrated. Devoid of station-keeping ability, space debris and fuel-depleted satellites often enter uncontrolled tumbles on-orbit. In order to perform on-orbit servicing or active debris removal, docking spacecraft (the “Chaser”) must account for the tumbling motion of these targets (the “Target”), which is oftentimes not known *a priori*. Accounting for the tumbling dynamics of the Target, the Chaser spacecraft must have an algorithmic approach to identifying the state of the Target’s tumble, then use this information to produce useful motion planning and control. Furthermore, careful consideration of the inherent uncertainty of any maneuvers must be accounted for in order to provide guarantees on system performance. This study proposes the complete pipeline of rendezvous with such a Target, starting from a standoff estimation point to a mating point fixed in the rotating Target’s body frame. A novel visual estimation algorithm is applied using a 3D time-of-flight camera to perform remote standoff estimation of the Target’s rotational state and its principal axes of rotation. A novel motion planning algorithm is employed, making use of offline simulation of potential Target tumble types to produce a look-up table that is parsed on-orbit using the estimation data. This nonlinear programming-based algorithm accounts for known Target geometry and important practical constraints such as field of view requirements, producing a motion plan in the Target’s rotating body frame. Meanwhile, an uncertainty characterization method is demonstrated which propagates uncertainty in the Target’s tumble uncertainty to provide disturbance bounds on the motion plan’s reference trajectory in the inertial frame. Finally, this uncertainty bound is provided to a robust tube model predictive controller, which provides tube-based robustness guarantees on the system’s ability to follow the reference trajectory translationally. The combination and interfaces of these methods are shown, and some of the practical implications of their use on a planned demonstration on NASA’s Astrobee free-flyer are additionally discussed. Simulation results of each of the components individually and in a complete case study example of the full pipeline are presented as the study prepares to move toward demonstration on the International Space Station.

## 1 Introduction

Tumbling objects are commonplace on-orbit. Spent rocket bodies, fuel-exhausted satellites, and space debris are all examples of potential free-tumbling objects. In a variety of sub-fields of space robotics, including on-orbit servicing and repair, active debris removal, and on-orbit assembly, the ability to dock with arbitrary tumbling objects given the limited initial knowledge of the Target object is a key capability (Flores-Abad et al., 2014). Often, these tasks are safety critical but cannot allow human-in-the-loop oversight due to the complexity of the maneuvers involved and the lack of reliable teleoperation and communication. As a result, robotic autonomous execution of rendezvous activities is a desirable capability in order to repair satellites, de-orbit debris, and more.

Consequently, autonomous docking with tumbling Targets is an active area of literature in the space robotics community, with a variety of individual algorithmic contributions over the past two decades. Some of the earliest studies dealt with motion and parameter estimation of tumbling Targets, the first step in preparing for rendezvous (Hillenbrand and Lampariello, 2005). Assuming Target knowledge, multiple early studies also proposed a variety of motion planning and control techniques, including deterministic approximate analytical trajectory optimization (Fejzi and Miller, 2008), detailed optimal control formulations (Aghili, 2008), and nonlinear optimization-based formulations (Lampariello, 2010). Work in this area progressively added greater complexity, including increased sources of uncertainty (Aghili, 2012; Sternberg and Miller, 2018) and more advanced motion planning approaches (Aghili, 2020).

Recently, some work has begun to explore the significant robotics systems integration considerations required for the integration of multiple elements of the autonomous rendezvous phase: estimation, motion planning, and control under uncertainty, to name a few (Terán Espinoza et al., 2019). Leveraging recent work in robust control and planning, the ability to provide guarantees on system performance in the real-time setting of autonomous rendezvous has brought full-fledged autonomous docking frameworks within reach (Limon et al., 2008; Majumdar and Tedrake, 2017; Buckner and Lampariello, 2018; Mammarella et al., 2018). However, the need remains for a complete autonomy pipeline for such a maneuver that is additionally robust to the most significant uncertainty sources of autonomous docking with tumbling Targets and that can operate with automatic visual estimation and motion planning components.

This study details such a framework that can account for some of the key uncertainty sources of tumbling rendezvous; specifically, the unknown Target tumble state. This framework, currently scheduled for a series of International Space Station (ISS) tests in 2021, introduces the algorithmic approach to connect these submodules to make autonomous close proximity rendezvous a reality, while additionally considering uncertainty due to imperfect knowledge of these tumbling Targets. Furthermore, this study describes the unique way in which this form of uncertainty results in an uncertain reference trajectory and discusses the limits on providing robustness guarantees. Initial results on all parts of the autonomy pipeline are presented independently, along with a full case study example of an autonomous rendezvous in a detailed simulation environment.

The remainder of this article is formulated as follows: Section 2 outlines the autonomous rendezvous problem; Section 3 details the varied methods needed to form the full autonomy pipeline and how these segments interact; Section 4 presents results of individual components as well as a case study example of the full pipeline algorithm in a detailed simulation environment; and finally, Section 5 includes a discussion of the proposed pipeline and plans for integration and future experimental testing.

## 2 Problem Formulation

The problem considers a close proximity rendezvous maneuver within the last ∼20–40 m of the approach operation, with the goal of safely approaching a tumbling free-floating object (the “Target”) and reaching a predefined offset point fixed in the Target’s body frame called the mating point (MP). It is assumed that the Target is non-cooperative and passive. The Chaser spacecraft that will perform the rendezvous begins at some initial standoff offset distance $r_{\text{off}}$ and is equipped to perform visual estimation of the Target. Geometric and velocity state constraints X on the Chaser motion are given by the geometry and motion of the Target and the velocity and operational limits of the Chaser. The Chaser also has input limits U due to its thruster capability. The Chaser has rough knowledge of the Target’s inertial parameters, but not its spin. A strategy is required to perform ingress to the uncertain tumbling Target and sync up with a safe and predefined MP. Ideally, any motion planning will be optimal with respect to some performance measure and maintaining some level of robust execution with respect to any plan is desirable.

### 2.1 Representative Satellite System of Interest

The Astrobee robots are a series of free-flying robots operating aboard the ISS with the purpose of 1) astronaut assistance and 2) microgravity autonomy research (Albee, Ekal, and Oestreich, 2020). In this way, the Astrobee program serves as a successor to the Synchronized Position Hold, Engage, and Reorient Experimental Satellites (SPHERES) in terms of guidance, navigation, and control experiments in microgravity Saenz-Otero and Miller (2005). The development of the autonomy pipeline proposed in this study has been produced in conjunction with scheduled ISS testing on the Astrobee hardware, and so uses this representative satellite system for demonstration purposes.

Astrobee is one of the first reusable microgravity testbeds capable of providing the hardware needed to test an autonomous tumbling rendezvous framework. Astrobee utilizes two impellers which are throttled in each direction to provide full holonomic propulsion capability. The robots use multiple sensors for navigation, including 2D visual cameras, 3D time-of-flight (ToF) cameras, and an inertial measurement unit (IMU). The flight software is implemented on two general-purpose processors, each running Ubuntu 16.04 and the Robotic Operating System (ROS). Two Astrobees are used in this experiment: one designated as the Chaser satellite, and the other designated as the Target object. The Chaser’s forward-facing 3D ToF camera (“HazCam”) and IMU provide the front-end measurements to the relative state and parameter estimation problem.

### 2.2 System Dynamics

Two sets of dynamics are at work: those of a partially uncharacterized/uncertain tumbling rigid body Target and of a rigid body Chaser. The Newton–Euler equations can be used to fully describe the 6 degree of freedom (DOF) motion of both systems. The translational dynamics are of particular interest for the Chaser with respect to motion robustness (i.e., collision avoidance).

#### 2.2.1 Reference Frames

The frames of reference used in this study are defined as follows:

T:Target Body Frame (centred at the centre of mass of the Target Satellite),

C:Chaser Body Frame (centred at the centre of mass of the Chaser Satellite),

G:Geometric Frame (arbitrary fixed body frame on the surface of the Target),

W:Inertial Frame of the International Space Station.

**FIGURE 1:**
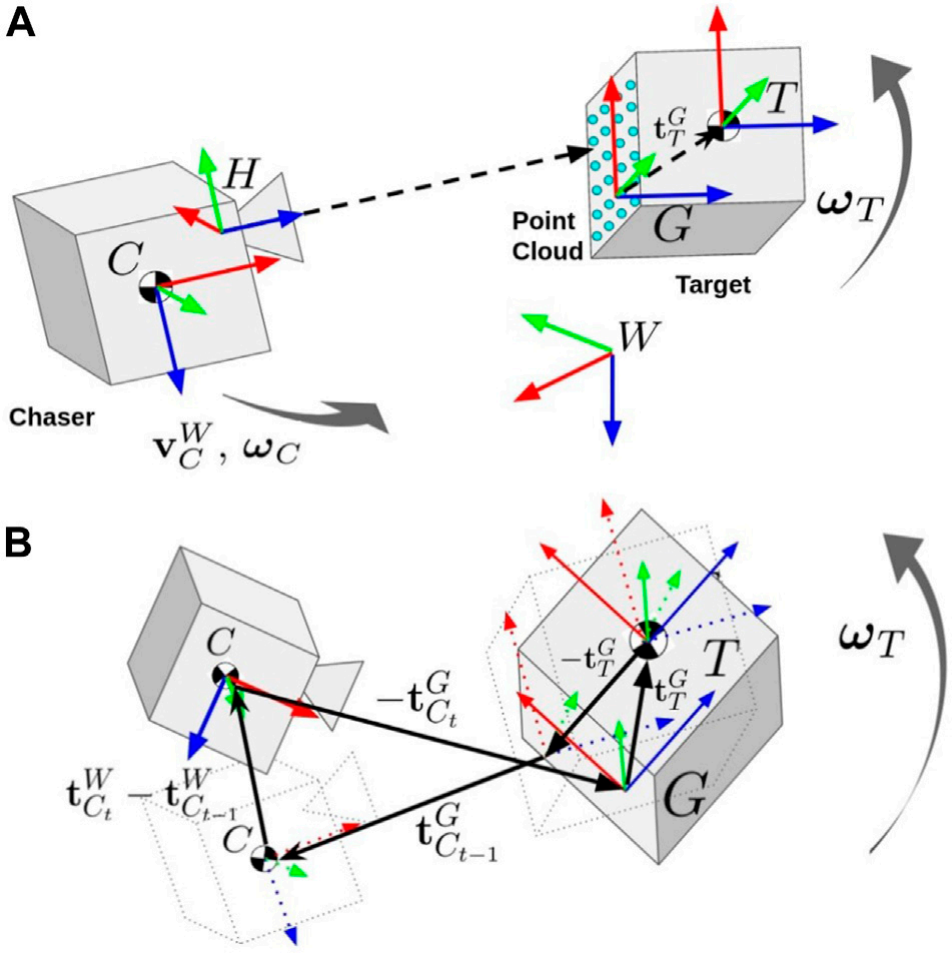
**(A)** The relevant coordinate frames for estimation of the Chaser and Target states and Target inertial parameters. The Chaser’s body frame C moves while the 3D ToF camera (frame H) collects point cloud measurements of the Target. The Target’s body frame (T) tumbles around its center of mass. The geometric frame (G) origin is defined at the centroid of the first point cloud measurement with an orientation matching the Target’s body frame. There is a constant translation vector $t_{T}^{G}$ between the Target’s body frame and geometric frame which must be estimated. **(B)** The rotation kinematic factors that link the otherwise separate Target and Chaser pose chains in the factor graph formulation are also depicted. The bold vectors in the image must sum to zero to satisfy the factor’s constraint.

These are depicted in context of the relative state estimation problem in Figure 1. Note that frames are also referred to using ℱT notation, and left superscripts indicate the frame of a vector quantity when the frame is not obvious.

#### 2.2.2 Tumbling Target Rigid Body Dynamics

The Target is assumed to be a rigid body in a free tumble, the dynamics of which are described by the Newton–Euler equations. The full equations of motion are given by the following:

$$\begin{array}{cc} \begin{array}{l} r≜{\left[\begin{array}{ccc} r_{x} & r_{y} & r_{z} \end{array}\right]}^{⊤}\\ q≜{\left[\begin{array}{cccc} q_{x} & q_{y} & q_{z} & q_{\theta } \end{array}\right]}^{⊤}\\ v≜{\left[\begin{array}{ccc} v_{x} & v_{y} & v_{z} \end{array}\right]}^{⊤}\\ \omega ≜{\left[\begin{array}{ccc} \omega_{x} & \omega_{y} & \omega_{z} \end{array}\right]}^{⊤} \end{array} & \;\;x≜\left[\begin{array}{c} r\\ q\\ v\\ \omega \end{array}\right] \end{array},$$ (1)

$${\dot{r}}_{CoM}=v,$$ (2)

$${\dot{v}}_{CoM}=\frac{F}{m},$$

$$\dot{\omega }=-I^{-1}\omega \times I\omega +I^{-1}\tau ,$$

q˙TI=12H¯(qTI)⊤ωIBT,

where the rotational terms are of interest, namely, ω and qTI describing the Target’s angular velocity and attitude with respect to the local orbital (inertial) frame W, respectively. The input torques τ are set to zero for the free-tumbling scenario of interest. The inertia tensor I is given fully as:

$$I≜\left[\begin{array}{ccc} I_{xx} & I_{xy} & I_{xz}\\ I_{yx} & I_{yy} & I_{yz}\\ I_{zx} & I_{zy} & I_{zz} \end{array}\right],$$ (3)

where the products of inertia $I_{xy}$, $I_{xz}$, and $I_{yz}$ are 0 in a principal axes frame. In order to solve the initial value problem (IVP) of future Target states for an inertia tensor in any arbitrary Target frame, it is necessary to know $q\left(t_{0}\right),\omega \left(t_{0}\right)$, and I.

The inertia tensor and initial angular velocity can yield a large variety of future system behaviors. A classic example is the flat spin, where ω is aligned with the maximum moment of inertia axis. In this case, the tumble is simply one-dimensional about this axis. In a more extreme case, the angular velocity changes drastically and suddenly when aligned with an intermediate moment of inertia axis, flipping approximately one and a half revolutions from one axis alignment to the other (Harris, 1994).

The Target is free to tumble, and dissipative effects are considered negligible over the timescales of rendezvous. Uncertainty in the Target’s tumbling dynamics will result in an uncertain solution to the IVP, addressed in Section 2.4.

#### 2.2.3 Chaser Translational Dynamics

The Chaser is also a 6 DOF rigid body and obeys the same set of dynamics as the Target, albeit with its own unique parameters. Since the translational dynamics of interest are purely linear they may be written in state space form, as follows:

$$z^{+}=\mathrm{Az}+\mathrm{Bu},$$ (4)

$$A=\left[\begin{array}{cccccc} 1 & 0 & 0 & dt & 0 & 0\\ 0 & 1 & 0 & 0 & dt & 0\\ 0 & 0 & 1 & 0 & 0 & dt\\ 0 & 0 & 0 & 1 & 0 & 0\\ 0 & 0 & 0 & 0 & 1 & 0\\ 0 & 0 & 0 & 0 & 0 & 1 \end{array}\right],$$ (5)

$$B=\left[\begin{array}{ccc} \frac{dt^{2}}{2m} & 0 & 0\\ 0 & \frac{dt^{2}}{2m} & 0\\ 0 & 0 & \frac{dt^{2}}{2m}\\ \frac{dt}{m} & 0 & \\ 0 & \frac{dt}{m} & 0\\ 0 & 0 & \frac{dt}{m} \end{array}\right],$$ (6)

$$z={\left[\begin{array}{cccccc} r_{1} & r_{2} & r_{3} & v_{1} & v_{2} & v_{3} \end{array}\right]}^{⊤},$$ (7)

where z indicates a nominal state which is derived from a deterministic dynamics model of the Chaser (${ℳ}_{C}$), $z^{+}$ indicates a discrete state update, and dt is the timestep. Note that orbital dynamics effects (e.g., the Clohessy–Wiltshire equations) are not considered in this particular problem statement due to the short timescales and distances under consideration in the system of interest (1.5 meters and 3 minutes). However, the linear CHW dynamics could easily be substituted into the system dynamics models of multiple components of the pipeline detailed in subsequent sections, which is intended for use in the range of ∼20–40 m of the Target, and with motion plan timescales of up to approximately 10 [minutes], as demonstrated in Stoneman and Lampariello (2016). If desired, the CHW equations are additionally applicable for distances up to a few kilometers Fehse (2003). However, the control forces at play noted in Section 2.3 are dominating with respect to any orbital mechanics or perturbation forces.

The Chaser and Target Astrobees each have a mass of 9.58 kg and inertial parameters $I_{xx}=0.153$, $I_{yy}=0.143$, and $I_{zz}=0.162\;\text{kg}\;{\text{m}}^{2}$. The robots are capable of providing up to 0.4 N of force and up to 0.04 Nm of torque. Additionally, the tumbling motion of the Envisat satellite is of interest ESA (2015). The inertial parameters of Envisat are also provided in the Envisat body frame:

$$I_{ES}=\left[\begin{array}{ccc} 17023.3 & 397.1 & -2171.4\\ 397.1 & 124825.7 & 344.2\\ -2171.4 & 344.2 & 129112.2 \end{array}\right]\;\left[\text{kg}/{\text{m}}^{2}\right].$$ (8)

The Envisat tumble can be mimicked by an actively controlled Astrobee with a different inertia tensor—precomputed tumble types can be actively tracked to simulate the tumble of a different object.

### 2.3 Chaser Motion Constraints

As it is typical in on-orbit missions, the maneuver is constrained by the Target geometry, as well as the Chaser’s limits on velocity and input. Constraints are additionally drawn from safety considerations including collision avoidance and plume impingement of the Target. The thruster authority available to the Chaser from the on-board thrusters dictates the available Chaser linear force and rotational torque.

#### 2.3.1 Constraints for the System of Interest

For the particular scenario of interest involving Astrobee, the motion constraints also result from the restrictions on Chaser movement dictated by the space available in the ISS Japanese Experiment Module (JEM) which houses the Astrobee platform, resulting in additional position and velocity constraints on the Chaser.

Considering that the approach tasks are restricted to the JEM, the robots are confined to move within a workspace of dimensions of approximately 1.4×2.8×3.4 m. The Astrobee specifications indicate that robots are restricted to move at a rate of 0.1 m/s in each spatial dimension, and the thrusters can provide forces $f=[f_{x},f_{y},f_{z}{]}^{⊤}=[0.452,0.216,0.257{]}^{⊤}$ N and torques $\tau =\left[\tau_{x},\tau_{y},\tau_{z}\right]$ ^T^ Nm approximately equal to one-tenth of the force available in each spatial dimension (NASA, 2015).

**FIGURE 2:**
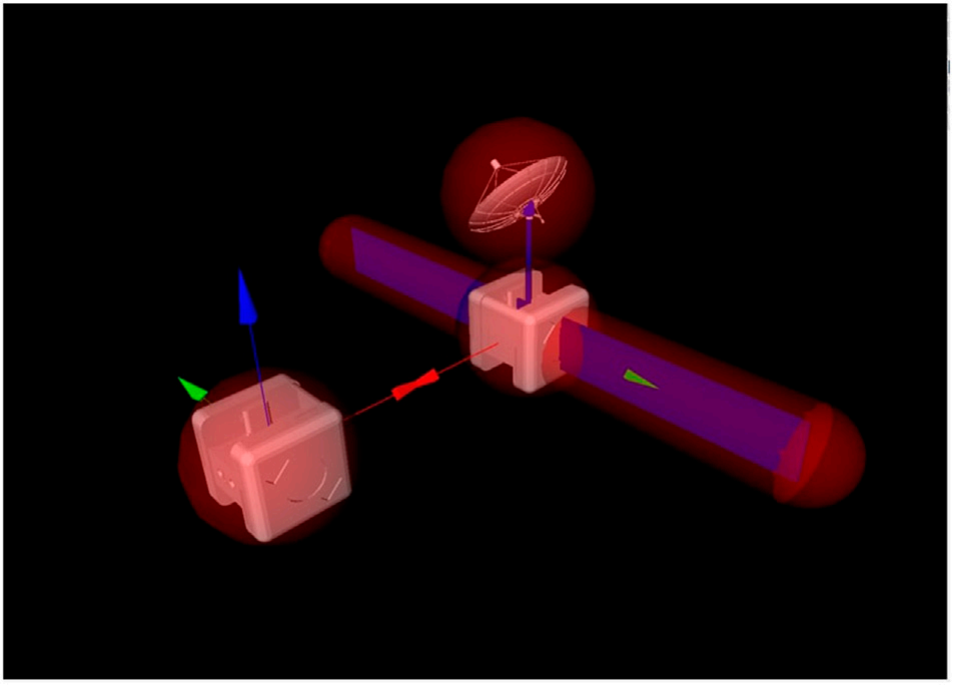
Scene depicting the approach of the Chaser to the Target Astrobee. The virtual geometry of the Target Astrobee is shown.

While the Astrobee robots have a fairly simple shape, the inertia of the Target satellite has been simulated, as discussed in the previous section, to match the more complex shape of the Envisat satellite. It is further assumed that this complex shape is the actual shape of the Target spacecraft. The Target Astrobee robot, therefore, wears a virtual geometry, similar to the real geometry of Envisat. As can be seen in Figure 2, the virtual geometry of the Target Astrobee includes a large solar panel and antenna appendages. This makes the collision avoidance task non-trivial. This complexity is tackled by creating a geometric model (ℳ) for each of the Chaser and Target shapes and using these to conduct collision detection. This geometric model appears in Figure 2 as translucent red shapes encapsulating the Target and Chaser geometries. The penetration depth (*d*) is then used to formulate the collision avoidance constraint (see Section 3.3). Figure 2 additionally shows how the x-axis of the Chaser body frame is required to point toward the center of mass of the Target robot, a field of view constraint. The Chaser must, therefore, rotate its attitude as well as translate in the course of the approach maneuver. This finally indicates that a torque constraint is present in tandem with the force limitations, limiting how fast the Chaser can reorient itself to maintain the field of view constraint.

### 2.4 Uncertainty Sources

Uncertainty enters the problem in a unique fashion: normally, additive uncertainty is thought of as a representation of unknowns in a system’s dynamics, environmental disturbances, and “noise.” However, in this problem the uncertainty of interest is due primarily to the reference trajectory itself (Buckner and Lampariello, 2018). As the motion planner must use the predicted Target motion to successfully reach the MP and avoid collisions, deviations in the Target motion due to parameter uncertainty in the Target’s initial tumbling state must be accounted for.

First, an approach trajectory in the Target’s body frame xrefT(k=0,1,… N) is provided by a motion planner, guaranteeing collision avoidance. This trajectory is rotated into the inertial frame using the nominal predicted Target attitude R¯TI(k) at each timestep, as given below:

xrefI(k)=TIR¯(q¯TI(k),ω¯IT(k),I¯)Txref(k), (9)

which is a function of the initial Target state estimate (q¯TI(0),ω¯IT(0)), inertial parameter estimates or prior knowledge $\bar{I}$, and the tumbling dynamics.

The nominal inertial trajectory xref,0I(k=0,1,…N) provided by the motion planner guarantees feasibility and collision avoidance based on the nominal predicted Target motion. However, this predicted motion is subject to initial estimation errors and Target inertia parameter uncertainty. As the controller performs the approach maneuver, the real collision-free trajectory in the inertial frame

xrefI(k)=TIR^(qTI(k),ωIT(k),I)Txref(k) (10)

will differ from the nominal trajectory due to the real Target motion. This creates an additive disturbance to the system in the inertial frame dependent on initial estimate inaccuracy, the inertial parameter estimates or prior knowledge, and the rate and accuracy of online updates,

wI(k)=xrefI(k)−xref,0I(k). (11)

As such, the controller should strive to track xrefI(k) in order to maintain the guarantees of the nominal body frame trajectory. However, for high levels of wI(k), the Chaser’s available actuation will not be capable enough to stabilize this disturbance, thus removing robustness guarantees from the docking trajectory—this is an interesting case noted in Section 3.5.

## 3 Approach and Methods

### 3.1 The Autonomy Pipeline: Concept of Operations

**FIGURE 3:**
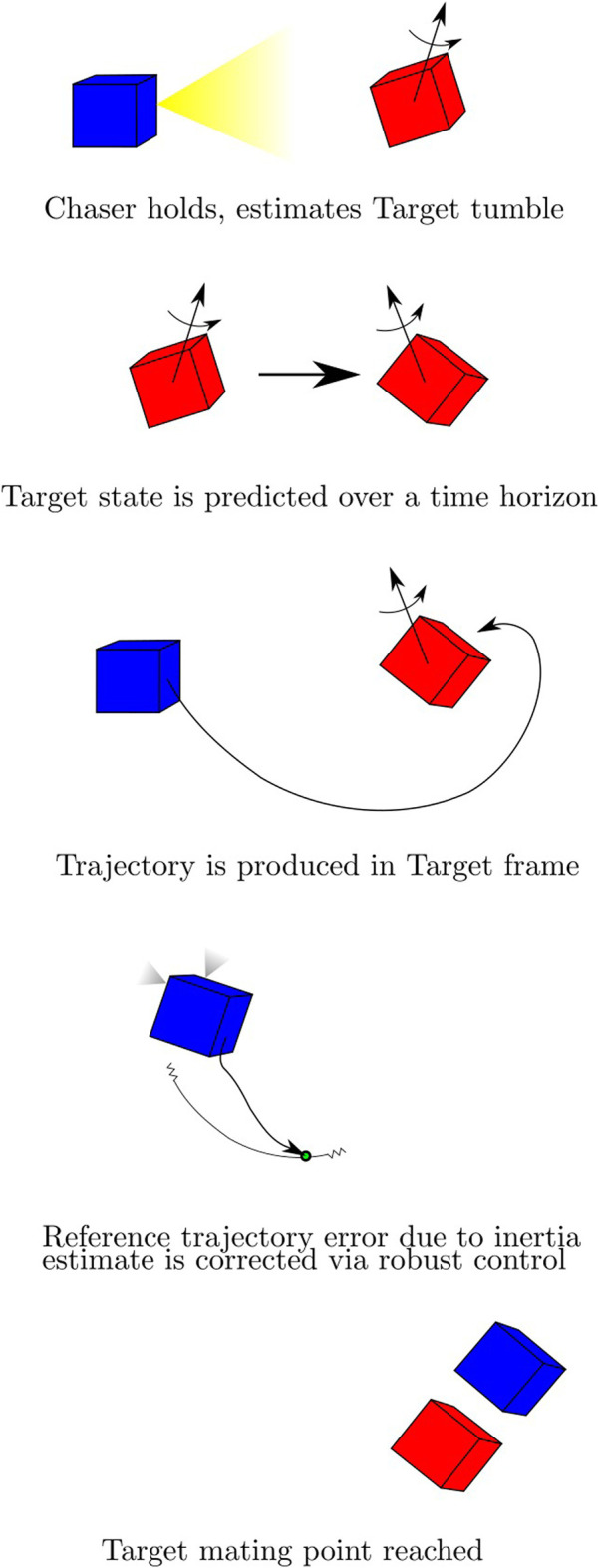
The concept of operations for the autonomous docking procedure with Chaser (blue) and Target (red). Following a standoff estimation phase a motion planner uses an estimate of future Target attitude to create a plan in the inertial frame, which is tracked by a robust controller. Uncertainty is converted from Target parameter uncertainty to Chaser reference trajectory uncertainty bounds.

**FIGURE 4:**
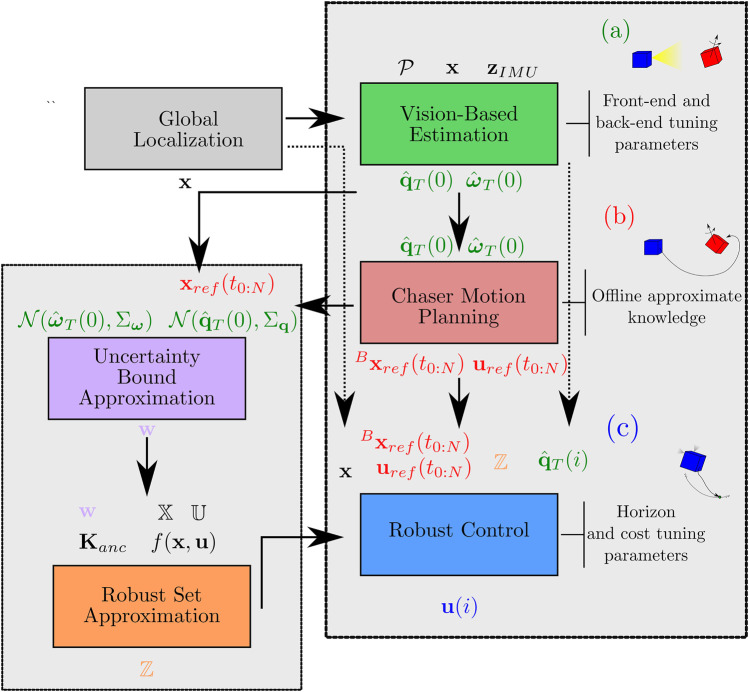
The full algorithmic pipeline, showing major inputs and outputs of each component algorithm. Inputs are shown above, and outputs are shown below. **(A)** shows the SLAM and polhode analysis visual estimation of the Target, **(B)** shows the online motion planning component, and **(C)** shows the robust model predictive controller and its supporting algorithms.

The approach taken can be broken into several distinct phases, depicted pictorially in Figure 3. First, the Chaser vehicle uses its time in the holding standoff position within approximately ∼20–40 m of the Target to gather observations of the Target tumble state and produce estimates of its angular velocity and attitude as well as its principal axes of rotation, as discussed in Section 3.2. Next, the Target tumble may be propagated using the nominal Target model, enabling the motion planning process described in Section 3.3 and the calculation of an uncertainty bound using the statistics of these estimates, as in Section 3.4. In Section 3.5, the maneuver is executed using tube-based robust model predictive control to retain performance guarantees. Finally, the MP is reached and the mission concludes. The algorithmic components in each of these phases are depicted in Figure 4, delineating the flow of inputs and outputs to each component of the algorithm.

### 3.2 Relative Navigation and Target Characterization

As outlined in Section 3.1, on-orbit inspection of an unknown spacecraft is not only a required component of currently proposed missions (Sullivan et al., 2015) but also of the more general spacecraft close-proximity operations (Terán Espinoza et al., 2019). There have been numerous research efforts to implement simultaneous localization and mapping (SLAM) approaches for estimating the Chaser and Target states as well as the Target’s inertial parameters (Tweddle et al., 2015; Setterfield et al., 2018a; Terán Espinoza, 2020). These studies utilize incremental smoothing and mapping (iSAM) (Kaess et al., 2008; Kaess et al., 2012) with stereo camera and IMU measurements to recover both the inspector and the Target state estimates in real time. Microgravity experiments on the ISS-based SPHERES testbed indicated the success and potential of these algorithms (Fourie et al., 2014; Tweddle et al., 2016). As such, this pipeline adapts the framework of Terán Espinoza (2020) for the inspection of an unknown tumbling Target using Astrobee’s sensor suite to provide SLAM front-end measurements. The full SLAM framework is implemented in Astrobee’s simulation environment via ROS.

The remainder of this section describes the specific details of the portion of the scenario handled herein (e.g., sensor suite), the mathematical formulation used to formalize the problem (factor graphs), the front-end components developed for information extraction (e.g., point cloud registration), the back-end components used for sensor fusion (e.g., iSAM), and the accompanying problems resolved in incorporation with the pipeline.

#### 3.2.1 Scenario Description

It is assumed that the Chaser satellite has a sensor suite capable of providing internal IMU measurements and external measurements that can be interpreted by a front-end as the Target pose. The Chaser Astrobee is equipped with the following sensors that fulfill these requirements: an IMU for ego-motion estimation, received at 62.5 Hz; and a 3D time-of-flight (ToF) camera to return 3D information of the Target for feature extraction and tracking purposes, received at 5 Hz. The 3D ToF camera is forward-facing on Astrobee and has a resolution of 224×171 pixels.

Using the acquired per-frame IMU and 3D depth information, the goal is to incrementally estimate the dynamic states of the Target and Chaser.

Target :(TTW,ωTT),

Chaser:(TCW,vCW,ωCC),
where T represents complete pose (position and attitude) information. The problem is formulated as a factor graph SLAM problem using odometry measurements from the ToF camera and the Chaser’s IMU. An overview of relevant coordinate frames for the SLAM problem is given in Figure 1.

#### 3.2.2 Factor Graphs and Problem Formulation

**FIGURE 5:**
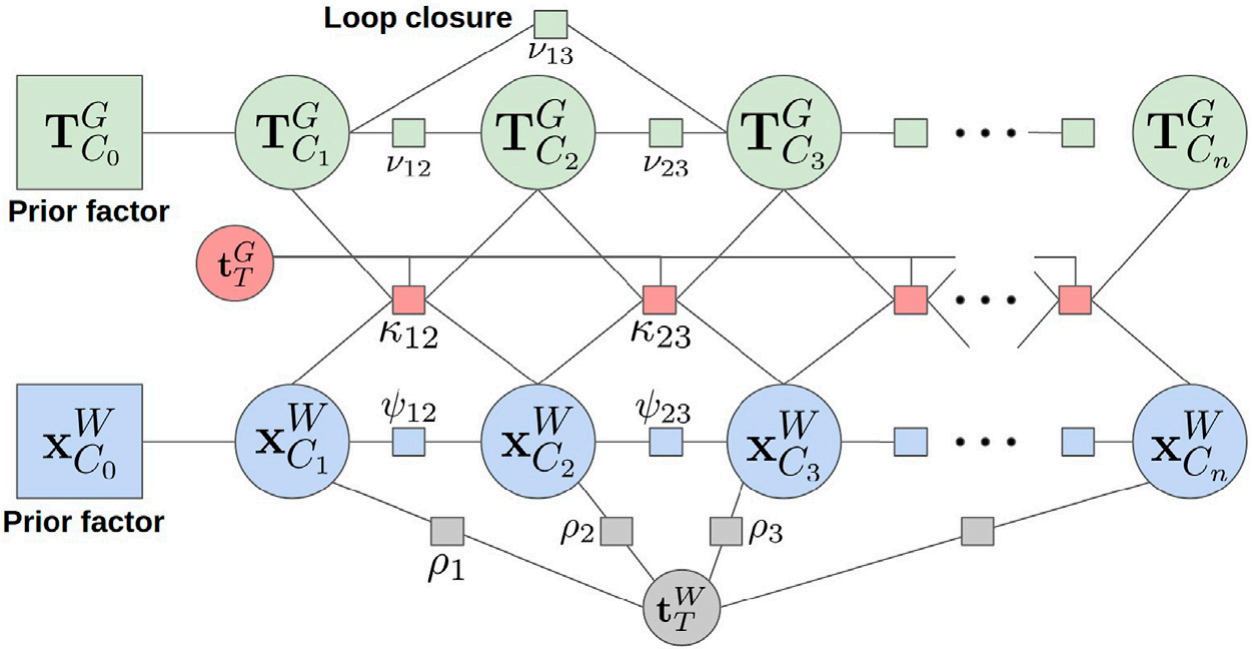
The full factor graph used for Chaser and Target state estimation. Nodes represent pose variables and are connected by ToF camera pose odometry, IMU odometry, range/bearing measurements, and rotation kinematic factors.

A bipartite factor graph connects all relevant variables and measurement factors for the relative state estimation problem (Figure 5). The graph is composed of independent Target and Chaser pose chains. Nodes represent unknown variables (the Chaser and the Target poses) and are connected by probabilistic factors that arise from odometry measurements. The Target motion TCnG is measured using a depth-based odometry, while the Chaser motion TCnW is measured using an IMU-based odometry. The two chains are connected via rotational kinematic factors that disambiguate the Chaser and Target’s motion, even while the Chaser is moving. Each measurement is associated with a predefined noise model based on the sensor’s characteristics. Through probabilistic inference on the factor graph, one can minimize the error associated with the factors and smooth all state estimates over time.

##### 3.2.2.1 Front-End Modules for Information Extraction

The Chaser’s ToF camera provides a 3D point cloud of the scene at regular intervals. This depth data provides measurements of the Target motion relative to the Chaser which are then used in the Target’s pose chain within the factor graph. The Teaser++ solver (Yang et al., 2020) is used to solve the point cloud registration problem between frames, thus providing pose odometry measurements with respect to the geometric frame ΔTCG(t−1;t). The geometric frame *G* is an arbitrary body-fixed frame on the Target with the origin placed at the centroid of the initial features gathered, and thus has some fixed offset from frame *T*. The registration problem is carried out via three steps: 1) detection of 3D features in the current frame, 2) matching of features between the previous and current frames, and 3) robustly solving the registration problem using the found matches. Fast point feature histograms (FPFH) are selected as the 3D feature descriptor for detecting and matching (Rusu et al., 2009). For a pair of point clouds $z_{i}^{\nu }$ and $z_{j}^{\nu }$ at timesteps *i* and *j*, the result is a pose odometry measurement

$$z_{ij}^{\nu }=\left\{z_{R_{ij}}^{\nu },z_{t_{ij}}^{\nu }\right\}\in SE\left(3\right),$$ (12)

of the Chaser with respect to the Target’s G frame.

The specific front-end for the Astrobee system of interest will be addressed here, though similar front-ends could be substituted for different satellite sensor suites. The Chaser’s IMU serves as the source of measurements for the Chaser’s pose chain in the factor graph. Since Astrobee’s IMU operates at a much faster rate than the ToF camera, the IMU preintegration theory from (Lupton and Sukkarieh, 2011; Carlone et al., 2014; Forster et al., 2016) is leveraged. The set of IMU readings $z_{{\text{IMU}}_{ij}}={\left\{z_{\omega_{\tau }},z_{a_{\tau }}\right\}}_{\tau =i}^{j}$ are bundled between keyframes at $t_{i}$ and $t_{j}$ as a relative transformation between the navigation states, allowing the collected information to be summarized as a single measurement of the following form:

$$\text{Δ}x_{ij}=\left\{\text{Δ}R_{ij},\text{Δ}t_{ij},\text{Δ}v_{ij},\text{Δ}b_{ij}\right\},$$ (13)

where R denotes the rotational part of the Chaser’s state, t the translation, v the velocity, and b the bias variables for the IMU.

Finally, range and bearing measurements of the Target are provided by the ToF camera point clouds. These measurements are defined as:

$$z_{j}^{\rho }=\left\{z_{j}^{\rho d}\in {\mathbb{R}}^{+},z_{j}^{\rho b}\in S^{2}\right\},$$ (14)

where $z^{\rho d}$ and $z^{\rho b}$ are assumed to be range and bearing measurements to the Target’s center of mass, respectively, with $S^{2}$ representing the two-sphere manifold.

##### 3.2.2.2 Factor Graphs for Probabilistic Inference

As stated above, the Target’s pose chain is established using features from the Target’s depth information, correlated across subsequent frames. The point cloud-based odometry factor *ν* added to the graph is formulated as a relative pose measurement constraint, as given below:

νij(TCiG,TCjG)∝exp{12‖(TCiGG⋅TCjG)⊖zijν‖Σνi2}, (15)

where $\|\left(\cdot \right){\|}_{\Sigma_{\nu }}^{2}$ is the weighted Mahalanobis distance using the noise model parameter $\Sigma_{\nu }$.

Loop closures on this pose chain can be implemented in the same manner by taking matching point cloud features across non-successive frames. Practically speaking, a randomly chosen past frame can be checked for loop closures with the current frame. If there are enough matches, the odometry is computed and added to the graph.

The Chaser’s pose chain is generated from IMU factors. To carry out inertial navigation using the IMU’s information, a binary IMU factor *ψ* is created of the form given below:

$$\psi_{ij}\left(x_{i},x_{j}\right)\propto \text{exp}\left\{-\frac{1}{2}\left\|\left(r_{\text{Δ}R_{ij}}^{⊤},r_{\text{Δ}t_{ij}}^{⊤},r_{\text{Δ}v_{ij}}^{⊤},r_{\text{Δ}b_{ij}}^{⊤}\right)\right\|\Sigma_{\psi_{ij}}\right\},$$ (16)

where r represents the residual errors for each of the terms shown in Eq. 13. This factor is placed between two subsequent navigation states $x_{i}$ and $x_{j}$ in the inertial navigation pose graph chain to constrain their relative motion using the IMU information.

The range and bearing measurements between the Chaser and the (approximate) Target’s center of mass are implemented as binary factors between the Chaser’s navigational state xCiW and a variable representing a constant Target’s center of mass offset tTW. Since the Target’s center of mass is assumed to be centered at the W frame origin, this extra variable simply accounts for any errors while modeling. The range and bearing factor is formulated as:

ρj∝exp{−12‖[hd(xCjW,tTW)−zjρdhb(xCjW,tTW)⊖zjρb]‖Σρ2}, (17)

where $h_{d}$ and $h_{b}$ are the measurement models for range and bearing.

Rotation kinematic constraints are used to disambiguate the motion of the Target and Chaser satellites. The rotation kinematic factor enforces a zero sum vector addition between temporally equivalent time step pairs in the Target and Chaser pose chains, given the assumption that the Target is unperturbed and translationally stationary. The rotation kinematic factor is built by following the vector addition shown in Figure 1, to give:

κt−1,t(xt−1,xt,TCt−1G,TCtG)∝exp{−12‖RCt−1WRCt−1⊤Gt−1(tCt−1/Gt−1Gt−1−tC/GG)+RCWRCt⊤Gt(tC/GG−tCt/GtGt)+(tCt/WW−tCt−1/WW)‖Σρt−12}. (18)

Notice that the rotation kinematic factor is dependent on estimating the constant offset between the geometric frame and Target’s body frame, tTG (3). This translation is added as a single unknown variable to the factor graph that is connected to all rotation kinematic factors. The full factor graph, including the Target’s pose chain, Chaser’s pose chain, and rotation kinematic factors is shown in Figure 5.

While the factor graph shown thus far exactly represents the mathematical formulation of the problem being solved, the actual online solution is computed using a different, but directly related, data structure called the Bayes tree (Kaess et al., 2010; Fourie et al., 2020; Terán Espinoza, 2020). By recycling computations at each time step, a full SLAM solution can be computed fast and accurately every time new information is received.

#### 3.2.3 Estimation of Angular Velocity and Target Principal Axes

The above factor graph formulation allows the Chaser to estimate its own navigational state and the Target’s attitude at each timestep. These estimates are then processed to provide angular velocity measurements of each spacecraft and also determine the Target’s principal axes of rotation.

The instantaneous angular velocity estimates ωGG and ωCC are extracted at each timestep via the relative rotation between temporally subsequent poses in the world frame. Using the factor graph variables,

ωCiC=1ΔtijLog((RCiW)⊤RCjW), (19)

ωGiG=1ΔtijLog((RTiW(RTiG)⊤)RTi⊤W(RTiG)⊤), (20)

where Log acts as shorthand for the SO(3) logarithmic map log followed by the inverse skew symmetric matrix operator.

Note that the Target’s orientation and angular velocity estimates are with respect to the arbitrarily initialized G frame. While the tTG variable is solved for in the factor graph, the orientation RTG is still undetermined. This issue is resolved by estimating the principal axes of the Target’s inertia tensor and establishing a non-arbitrary *T* frame. Through polhode analysis,^1^ by Setterfield et al. (2018b), this misaligned angular velocity vector in the G frame is rotated such that the polhode’s new orientation produces central conic projections onto the XY, YZ, and XZ planes.

The specific combination of conic types for each plane further specifies the convention and estimate for the principal axes orientation. For instance, a tumbling Target with a tri-axial inertia tensor and low rotational energy will result in two ellipses and one hyperbola Setterfield et al. (2018b). The final result is an optimized orientation RPG that best aligns the measured angular velocity values into the newly defined P frame based upon the Target’s principal axes of inertia. The rotation between the principal axes frame and the defined Target frame T where Envisat’s inertia tensor is defined, RPT, is now used to determine the Target’s orientation and angular velocity with respect to the T frame, as given below:

RWT=RCW(RCG)⊤RPG(RPT)⊤, (21a)

ωTT=(RPG)(RPT)⊤ωGG. (21b)

Once the angular velocity profile has been correctly aligned with the principal axes, it is possible to proceed with the estimation of the Target’s inertia ratios if they are not already known. By leveraging the closed-form solution for rigid body motion based on the Jacobi elliptic functions Hurtado and Satak (2011), a second procedure from Setterfield et al. (2018b) can be employed to create a constrained nonlinear optimization problem and solve for physically consistent values for the inertia ratios $J_{1}$ and $J_{2}$. An alternative approach for identifying the inertia parameters, as well as the state parameters of a tumbling Target, can be found in Lampariello et al. (2021). The same study also presents a method for performing long-term motion prediction, while accounting for the state and inertial parameter dispersity which results from the parameter identification. This allows for robust motion prediction of the Target’s tumbling motion even if there is no available inertia tensor. The inclusion of such methods in the pipeline proposed here is a subject of future study.

### 3.3 Chaser Motion Planning

The motion planner considers only the nominal motion of the Target (propagated within the motion planner using Boost (Mascellani, 2019)) to generate nominal trajectories to control the Chaser. The method is based on Stoneman and Lampariello (2016), in which it is made evident that, if present, large appendages play an important role in the motion planning task. Similar approaches can be found in the literature, such as (Ventura et al., 2015; Park et al., 2017; Sternberg and Miller, 2018). In these studies, the convex optimization-based approach does not retain convergence guarantees. In Virgili-Llop et al. (2019), a covexification of the nonlinear collision avoidance constraint allows fast online planning for any relevant tumbling state of the Target, while retaining convergence guarantees. It is noted, however, that the method therein is based on a three-stage planning approach, necessary to account for the case in which the MP is inside the convex hull, as well as on repeated replanning, possibly for a complete period of motion of the Target, to identify the global minimum for the query at hand.

The Chaser trajectory $x_{ref}\left(p,t\right)$ is composed of three b-splines and their first three derivatives, one b-spline for each spatial dimension. Each spline has *n* free parameters **p** and is sampled at *m* via points. The Chaser pose $x_{ref}\left(t\right)$ considers the Chaser’s position and orientation such that $x_{ref}\left(p,t\right)=\left[x_{ref,p},x_{ref,o}]=[x_{ref,x},x_{ref,y},x_{ref,z},q_{ref,x},q_{ref,y},q_{ref,z},q_{ref,\theta }\right]$. At the final via point, the Chaser is required to meet the MP, matching the position and velocity of the MP in the inertial frame, and must be oriented such that the Chaser body frame’s positive x-axis points towards the Target’s center of mass.

The approach maneuver is formulated here as a nonlinear optimization problem (NLP) to ensure feasibility of the generated trajectories with respect to the motion constraints. The NLP minimizes the mechanical energy cost J(p), for a fixed final time $t_{f}$, and as a function of the free parameters of these b-splines subject to position, velocity, actuation, plume impingement, and collision avoidance constraints at each via point:

$$\underset{p}{\text{min}}J\left(p\right),$$ (22)

$$s.t.\;c_{position}\left(x_{ref,p}\left(p\right),t\right)\leq 0,$$

$$c_{velocity}\left({\dot{x}}_{ref,p}\left(p\right),t\right)\leq 0,$$

$$c_{force}\left({\ddot{x}}_{ref,p}\left(p\right),t\right)\leq 0,$$

$$c_{torque}\left(x_{ref}\left(p\right),{\dot{x}}_{ref}\left(p\right),{\ddot{x}}_{ref}\left(p\right),t\right)\leq 0,$$

$$c_{collision}\left(x_{ref,p}\left(p\right),t\right)\leq 0,$$

$$c_{plume}\left(r,q,x_{ref}\left(p\right),{\ddot{x}}_{ref,p}\left(p\right),t\right)\leq 0,$$

fort=0,…,m−1.

In this NLP, the first four constraints in the list are box constraints using the values outlined in Section 2.3. Additionally, X and U constraints are further tightened to provide a conservative bound on potential tightening required by the robust MPC nominal controller. It should also be noted that, while an optimal trajectory in the spatial dimensions is sought $x_{ref,p}\left(p,t\right)$, the orientation of the Chaser along this optimal trajectory must also be sought $x_{ref,o}\left(p,t\right)$, to ensure that its x-axis always points in the direction of the Target’s center of mass (field of view constraint). It can thence be determined if the necessary torque related to this pointing rotational motion is permitted by the Chaser thrusters.

Recalling the discussion of the collision avoidance constraint from Section 2.3, the collision constraint is given by the penetration depth *d* such that

$$c_{collision}\left(x_{ref,p}\left(p,t\right)\right)=d\left(q,x_{ref,p}\left(p\right),M,t\right)\leq 0,$$ (23)

which is an iterative, nonlinear function. Note that the Chaser is modeled as a sphere, therefore relieving the need to consider its orientation in this constraint. Geometric modeling and collision detection is implemented using the Open Dynamics Engine, and additionally tested using the Gazebo simulation environment, detailed in Section 4 (Smith, 2009).

In order to reduce the computation time, an online planning method has been devised which provides a warm start to the optimization problem. This method makes use of a precompiled look-up table (LUT) of corresponding pairs of initial conditions for the Target’s motion and composite optimization parameter solutions **p** for the three b-splines to identify a suitable initial guess for the planner. By inspecting the members of the LUT to select the initial guess, it is possible to quickly reach the local minimum in solving (Eq. 22).

This LUT must be generated offline, due to the computational burden involved. Its generation requires approximate knowledge of the inertia of the Target (up to a constant multiplying factor) and geometry of the Target. If the Target is initially entirely unknown with respect to these criteria, this missing information must first be identified and relayed to ground. The inertia can be identified using one of a variety of methods, including those detailed in Setterfield and Miller (2017); Teran, (2021); Lampariello et al. (2021) and the geometry can be determined using 3D reconstruction methods. However, aside from this modeling information (which is often given ahead of time, at least approximately) the motion planning method is entirely online-computable. The resulting NLP is implemented and solved using the SLSQP algorithm provided in the nonlinear optimization package NLopt (Johnson, 2017).

### 3.4 Uncertainty Bound Definition

**FIGURE 6:**
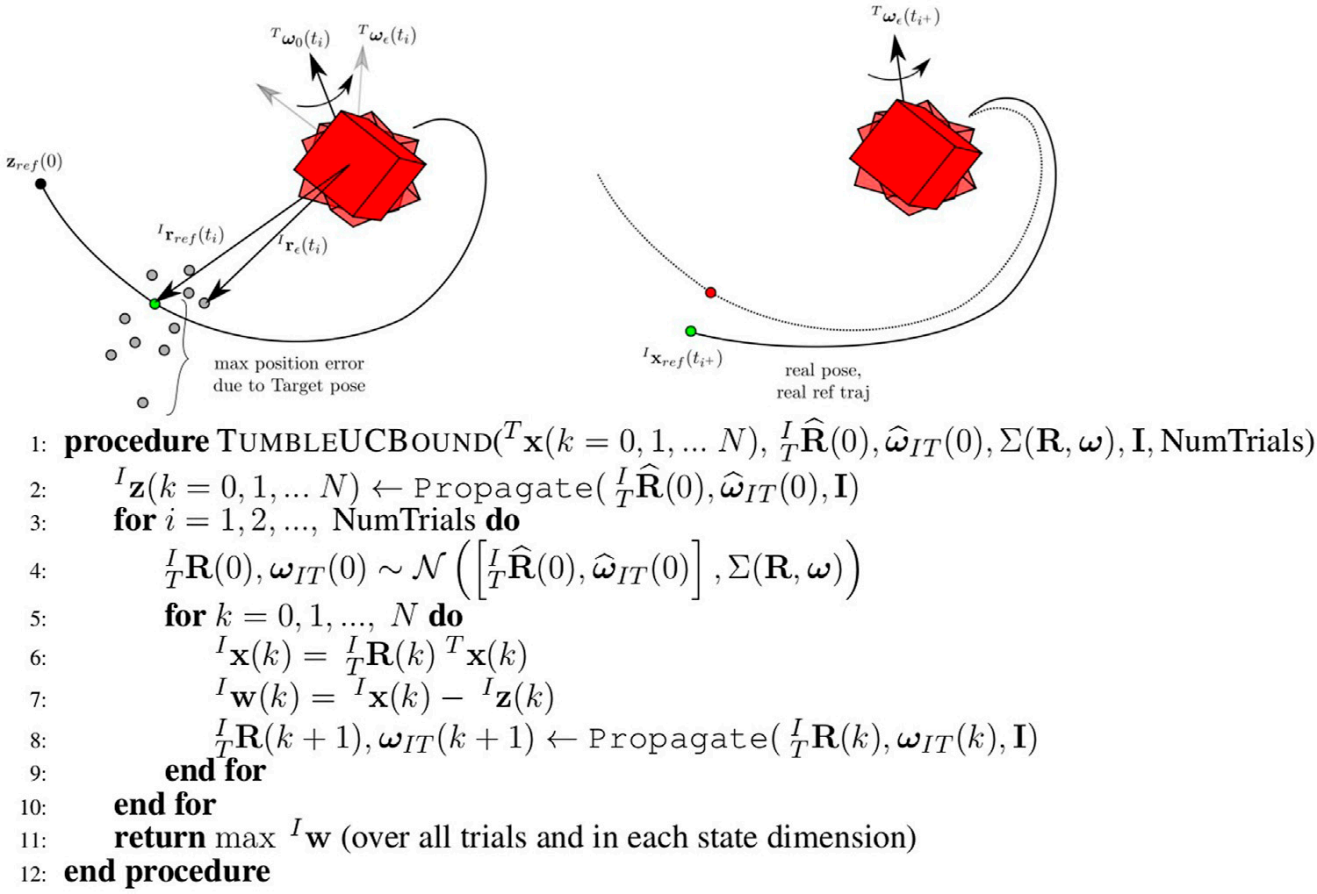
The Propagate step of the uncertainty bound procedure **(left)**. Many Monte Carlo runs over potential initial conditions and parameters for the Target result in many possible reference trajectory updates from $\left[t_{0},t_{f}\right]$. At each point in time, there is a maximum possible error from the nominal reference trajectory. A reference trajectory update resulting from a pose update **(right)**. When new Target pose estimates are available, xrefT can be repropagated in the inertial frame. The algorithm to compute the uncertainty bound for tube MPC is shown below. The maximum disturbance magnitudes are found over the course of a number of Monte Carlo trials.

The disturbance level of major uncertainty sources must be defined in order to provide a form of robustness guarantee. The tube model predictive control (MPC) relies on a known bound of the additive disturbances; in the context of the tumbling rendezvous maneuver, this means that limits must be determined for the magnitude of disturbances defined by Eq. 11, the primary uncertainty source of interest. Given a nominal reference trajectory provided by the motion planner and uncertainty levels of the Target state estimates a series of Monte Carlo trials is computed to approximate the maximum disturbance over the course of the trajectory in a manner similar to (Buckner and Lampariello, 2018). Additionally, the effect of online-updating (and the frequency of online updating) is accounted for in the approximation procedure. For each trial, the nominal initial Target state is perturbed within the state estimate uncertainty levels, based on the statistics of the state estimates. This creates a Target tumbling trajectory that differs from the predicted motion used by the motion planner. The disturbance is then measured at each timestep of the Monte Carlo trial trajectory by comparing the nominal reference trajectory to the “real” trajectory that arises from Eq 10. This difference constitutes the defined disturbance in Eq. 11. The repeated Monte Carlo trials build up a sampling of disturbance values seen across all trajectories and, like all Monte Carlo evaluations, increase the modeling accuracy of the true distribution with a greater number of trials. From there, the uncertainty bound is defined via the largest disturbance magnitudes in each state dimension. This is a conservative approximation, and can be relieved if a lower *σ* value is desired. The full algorithm to determine the uncertainty bound is detailed in the algorithm of Figure 6. Note that the current implementation assumes all disturbances arise from initial state estimate errors. This algorithm can be easily modified to include disturbances from inertia parameter error as well, if desired. The Monte Carlo uncertainty bound determination process is shown in Figure 6.

### 3.5 Robust Tube Model Predictive Control

Model predictive control (MPC) is a commonly used control technique which is particularly notable for its ability to approximately optimally control general dynamical systems under constraints. While proofs for MPC stability under certain conditions (Mayne et al., 2011) abound, guarantees for stochastic systems are generally lacking. Tube MPC is a notable exception, providing robustness guarantees when bounded additive uncertainty is encountered in the system dynamics. Using tube MPC, a portion of control authority is reserved for robust actuation, often in a simple feedback form to counter disturbances. The guarantee obtained is one of tube robustness—if a system starts in a tube of possible states, it remains within a tube around a nominal trajectory. These tubes are formed around the nominally planned MPC trajectory, and the motion of the system can be thought of as a composition of these planned “safe sets.”

Tube MPC methods exist for both nonlinear and linear systems with additive bounded uncertainty—for this particular problem linear tube MPC is of interest for the linear translational satellite (double integrator) dynamics. (Attitude control is not considered under this robustness paradigm: the Euler equations are nonlinear and pointing constraints under the tumbling uncertainty are not as important—line of sight constraints are somewhat generous and it is assumed that motion plans produce collision-free trajectories for a max-radius ball about the system’s center of mass.) In essence, two controllers must be produced: 1) a nominal MPC, operating under a modified set of constraints to account for worst-case uncertainty; 2) an ancillary or “disturbance rejection” controller that provides robustness to aleatoric uncertainty. Given a reference trajectory, tube MPC will repeatedly plan the nominal trajectory online, and append ancillary controller actuation to the system inputs, resulting in *tube robustness*.

**FIGURE 7:**
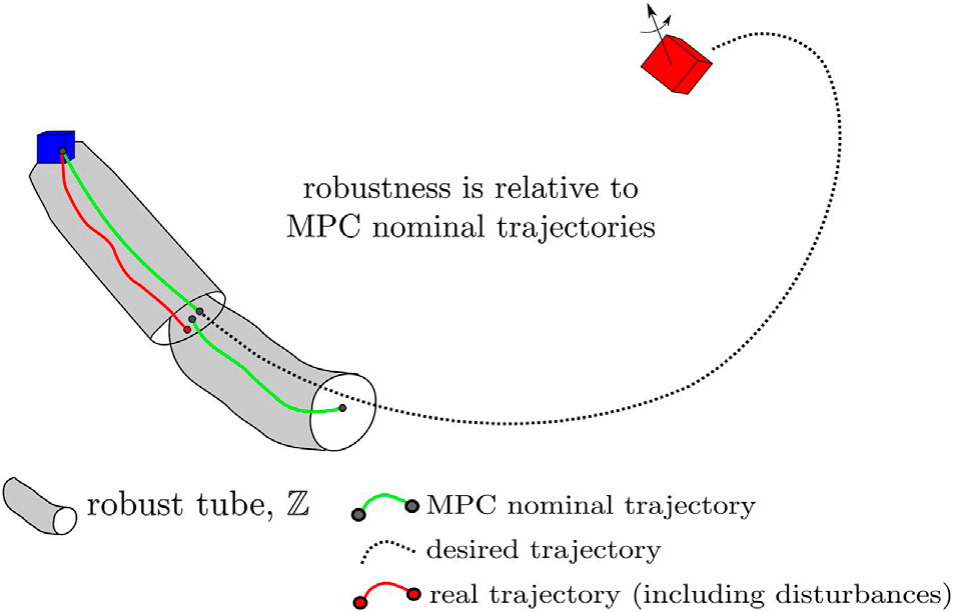
A demonstration of the tube robustness guarantee. Robust tubes are produced around nominal reference trajectories which need not necessarily coincide with the real initial state and are in turn made to track a motion planning reference trajectory, $x_{ref}$ (black). The maximum possible deviation of $x_{ref}$ in any given timestep is treated as an additive uncertainty for tracking, and is dealt with via the tube robustness guarantee.

This framework is now explained for the trajectory tracking of the uncertain rendezvous reference trajectory, requiring a few ingredients: a reference trajectory $x_{ref}\left(i\right)$, a reference input $u_{ref}\left(i\right)$, a Chaser dynamics model ${ℳ}_{C}$, and a Target dynamics model which includes parameter and angular velocity estimates and prior knowledge ${ℳ}_{T}:\left\{I,\hat{q},\hat{\omega }\right\}$ (for solving the IVP for pose propagation). The tube robustness guarantee for the robust rendezvous problem is depicted in Figure 7.

${ℳ}_{T}$, which is updated every time a pose observation is available, allows for continuous inertial frame updates of xrefI(i). Further, the algorithm of Figure 6 is used to convert the state Target uncertainty into a *predicted* bound on reference trajectory disturbance. The resulting Target orientation uncertainty is used to express an additive disturbance on the Chaser reference trajectory error dynamics, wI,

W={wI∈ℝn:[I6−I6]w≤[wmaxIwmaxI]}. (24)

The inertial frame Chaser translational error dynamics are then,

xerr+I=AxerrI+BIuerr+w, (25)

where $x_{err}=z-x_{ref,i}$ (where $x_{ref,i}$ is updated based on the latest pose estimate) and $u_{err}=u-u_{ref,i}$. w can be thought of as the worst possible disturbance in the reference trajectory that can be caused by a Target pose update at any given timestep.

The stochastic dynamics are now in a suitable format for linear robust tube MPC. First, a disturbance rejection controller called the ancillary controller must be used to reject disturbance from a nominal MPC trajectory. The ancillary controller takes the form below and is added onto a nominal MPC input, v:

$$u_{anc}=K_{\mathrm{anc}}\left(x-\bar{z}\right),$$ (26)

$$u=v+u_{anc},$$ (27)

where v is a nominal actuation determined by a deterministic MPC and $K_{anc}$ indicates the ancillary controller disturbance rejection gain, and $\bar{z}$ is a nominal state not necessarily the same as the real initial state. $K_{anc}$ can be determined through a simple LQR procedure on the nominal dynamics, but provides optimal performance and better robustness guarantees when determined via a tube minimization procedure (Buckner and Lampariello, 2018). That is, if the robust positively invariant set (RPI) can be minimized through the choice of $K_{anc}$ then more rigorous guarantees exist; namely, the tube robustness guarantee that if the system state x starts within a set ℤ centered around a planned control trajectory z, under the given uncertainty and ancillary controller it will remain with a tube around this trajectory for all possible w disturbances. The tube robustness guarantee can be thought of simply as “if you start in the tube, you stay in the tube.”

Secondarily, the actual nominal MPC trajectory z used by the ancillary controller must be determined. The error dynamics of Eq. 25 are used. U and X are converted to tightened constraints, effectively giving up control authority to the ancillary controller for disturbance rejection. These tightened constraints are indicated as $x\in \bar{X}\subset X$ and $u\in \bar{U}\subset U$ and are derived from the nominal box constraints, X and U. The exact constraint tightening procedure can be found in both Buckner and Limon (Limon et al., 2008; Buckner and Lampariello, 2018). A notable feature observed in this constraint tightening procedure is the fact that large uncertainty bounds will make constraint tightening infeasible. This serves as a notification that the considered uncertainty levels are beyond the system’s actuation and/or dynamics capability to adequately counter. It is possible to compromise and settle for a lower level of robustness with a lower *σ* for the procedure of Section 3.4, or to even require replanning at prior stages of the pipeline.

An additional modification to the MPC can be added: a parameterization of steady state values for the nominal system, captured in $\theta \in {\mathbb{R}}^{n_{\theta }}$ as defined in (Buckner and Lampariello, 2018). Finally, the nominal MPC (which does not necessarily align with the initial real state, $x_{i}$) can be found by solving:

$$\begin{array}{l} \underset{u\left(i\right),{\bar{z}}_{0},\bar{\theta }}{\text{min}}J=\sum_{i=0}^{N-1}{\left[{\bar{z}}_{i}-x_{i,ref}\right]}^{⊤}Q\left[{\bar{x}}_{i}-x_{i,ref}\right]+{\left[{\bar{v}}_{i}-u_{i,ref}\right]}^{⊤}R\left[{\bar{v}}_{i}-u_{i,ref}\right]+\\ {\left[\bar{z}\left(N\right)-x_{ref}\left(N\right)\right]}^{⊤}H\left[\bar{z}\left(N\right)-x_{des}\left(N\right)\right]+{\left[\bar{\theta }-\theta_{ref}\right]}^{⊤}T\left[\bar{\theta }-\theta_{ref}\right] \end{array}$$

subject to

$$z_{i}^{+}=f\left(z_{i},v_{i}\right).$$

$$\;\bar{z}\in \bar{X}$$

$$\;\bar{v}\in \bar{U}$$

$$\;\bar{v}\in x⊕\left(-\mathbb{Z}\right)$$

$$\;\left(\bar{z}\left(N\right),\bar{\theta }\right)\in \Omega^{e}$$

Eq. 27 is executed for the first timestep of the nominal MPC solution until the solution can be recomputed at $i^{+}$.

## 4 Results

The components of the full rendezvous pipeline were implemented and tested using Astrobee’s simulation environment. Astrobee’s software is based on the Robotic Operating System (ROS) framework, where modular software components (nodes) exchange messages over topics for complex multi-threaded code coordination. Each part of the proposed autonomy pipeline constitutes a ROS node that is added to Astrobee’s core flight software and in some cases overrides default behavior.

The Target coordinator provides torque-free tumbling motion setpoints that are tracked by a custom standard MPC controller. In this way, the Target Astrobee can follow correct tumbling trajectories for various inertia tensor test cases, such as Envisat’s. The Chaser coordinator orchestrates all parts of the autonomy pipeline and activates each step based on timing parameters and completion status. The full ROS architecture will be used in experimental testing on the ISS. Additional detail is provided in Section 4.4 on the full pipeline setup and testing.

Each component of the pipeline is first demonstrated against individual performance tests, in preparation for combined experimental testing. The full composition of algorithms is provided in Figure 4. The results of the vision-based state and parameter estimation are summarized in Section 4.1, the motion planning in Section 4.2, and the uncertainty bound and tube MPC in Section 4.3. In Section 4.4, a case study is presented for the full operations pipeline, providing a complete example of the full autonomous rendezvous pipeline operating in the high-fidelity Astrobee simulation environment.

### 4.1 State and Principal Axes Estimation Performance

In testing the relative state and principal axes estimation framework, the Chaser was initially situated 1.5 m away from the Target Astrobee, which had an initial angular velocity $\omega_{IT}\left(0\right)=\left[0,3.53,3.53\right]$ deg/s. In this case, the Target performed a tumble that abides by Envisat’s inertia tensor. The Chaser was commanded to follow a sample inspection trajectory consisting of 1) a lateral arc maneuver, 2) a vertical arc maneuver, and 3) an approach/recede maneuver along the viewing axis. Chaser attitude commands kept the Target in the HazCam field of view. The factor graph was solved at a rate of 1 [Hz]; most of the computation run-time was taken up by point cloud matching and registration, this fairly slow rate ensures that the factor graph updates are achievable on the robot hardware.

Downsampling and background elimination was applied to the point clouds for more efficient processing. Sufficient numbers of matches from frame to frame were found, which in turn enabled reliable pose registration solutions. In general, the Teaser++ pose registration solver provided robust pose odometry estimates of the Target’s geometric frame with low noise levels along with several loop closures. Truth values from the simulator serve as a metric to evaluate the proposed approach and provide estimation error statistics.

In terms of Chaser navigation, the estimates were smooth and closely corresponded to the true values, demonstrating the ability to disambiguate Chaser motion from the tumbling Target motion. The Target orientation and angular velocity estimates were estimated in the factor graph with respect to the arbitrary G frame. As such, there is a coordinate frame offset between the initially estimated values and the simulator truth values. These “unaligned” values were used to perform the polhode analysis in order to determine the principal axes of the Target and its inertia tensor ratios (if applicable).

**FIGURE 8:**
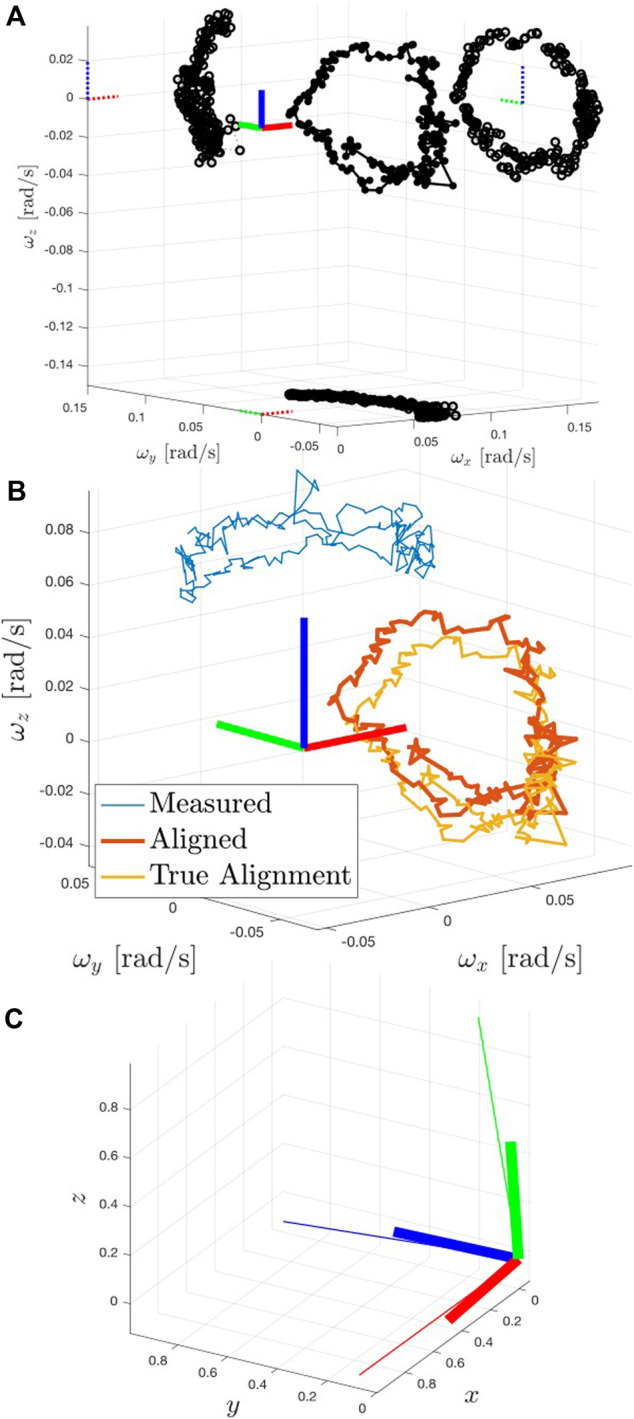
**(A)** Resulting polhode after alignment to the Target’s estimated principal axes. The polhode’s plane projections produce central conics (not rotated nor displaced from their respective origins). Solid circles indicate the polhode, and open circles indicate the projected conics. **(B)** Polhode comparison between the measured (blue, in geometric frame), aligned (red, after rotating by the estimated orientation from geometric to principal axes), and groundtruth-aligned (orange) curves. **(C)** Visual comparison of the offset between the groundtruth principal axes (bold RGB triad) against the estimated orientation (thin, longer RGB triad).

Figure 8 shows the results for the principal axes estimation portion of the pipeline. The optimized value of RPG produced the best conic projection fits shown in the figure. Since the Envisat’s inertia tensor is tri-axial, the projection of the polhode produced central ellipses in the XY and YZ planes, and a hyperbola in the XZ plane. This orientation is then used to rotate the “measured” Target angular velocity values (defined in the G frame) into the P frame.

Table 1 shares estimation error statistics for the navigational states. The low magnitude of these values indicate successful performance of the on-orbit inspection task, which in turn would enable the subsequent motion planning and trajectory tracking with online-updating phases that are required to intercept the Target.

**TABLE 1:** Estimation error statistics for the navigational states. All error values are the median L2 norm between the estimated and true values, except for $\tilde{a}$, which is computed as $\tilde{a}=Log\left({\bar{R}}^{⊤}\hat{R}\right)={\left[\delta a_{x},\delta a_{y},\delta a_{z}\right]}^{⊤}$ and $\tilde{\theta }=\left|\left|\tilde{a}\right|\right|$, where $\bar{R}$ and $\hat{R}$ correspond to true and estimated orientations, respectively.

${\tilde{t}}_{C}^{W}$	4.1 cm
${\tilde{v}}_{C}^{W}$	5.3 mm/s
${\tilde{a}}_{C}^{W}$, $\tilde{\theta }$	${\left[-0.1,0.001,0.61\right]}^{⊤}$, 1.00 deg
${\tilde{\omega }}_{C}$	0.08 deg/s
${\tilde{a}}_{T}^{W}$, $\tilde{\theta }$	${\left[6.26,-4.24,0.85\right]}^{⊤}$, 7.25 deg
${\tilde{\omega }}_{T}$	0.6199 deg/s

### 4.2 Chaser Motion Planning Performance

The motion planner was validated and analyzed for performance. A few statistics are note-worthy within the above context.

First, for the chosen tuning of the NLP implementation in terms of the cost function accuracy (1e-8) and constraint gradient finite-difference step size (1e-10) and with a warm start provided by the LUT and nominal initial conditions for the Chaser, it is found that the planner produces motion plans for random queries of the Target parameters with a mean runtime of 0.84 s with a 3σ of 2.03 s. Without this warm start, the mean planner runtime is 1.68 s and the 3σ is 5.56 s.

For 1,000 queries with a warm start of the motion planner and the Chaser located at the nominal initial position, there is a 100% success rate in the optimizer converging to a feasible trajectory. For comparison, for the same sample size and Chaser initial position, but with a cold start, the success rate is 90%.

As the Chaser will be placed at its initial position manually, the effect of placement error has also been investigated. For 10,000 samples of perturbation only in the x-direction of up to 10 cm, 10,000 samples of perturbation in the y-direction only of up to 10 cm, 10,000 samples of perturbation in the z-direction only of up to 10 cm, and 10,000 samples where the modulus of the perturbation in the x-, y-, and z-direction is up to 10 cm, the failure rate for the optimization to converge to a feasible trajectory is only 0.007%. When the initial conditions of the Chaser are within boundaries and a warm start is provided to the motion planner, the mean runtime is 0.92 s with a 3σ of 1.51 s.

### 4.3 Uncertainty Bound and Robust Tube MPC Performance

A reference trajectory $x_{ref}$ and reference input $u_{ref}$ were provided using the nonlinear programming-based motion planning method of Section 3.3. Using these references, the uncertainty bound method of Section 3.4 was used to produce W. Finally, using the tube MPC method of Section 3.5, an ancillary controller and nominal MPC were then created to robustly guide the Chaser along the reference trajectory in the inertial frame, with knowledge of the potential disturbances induced by inaccurate Target pose knowledge.

The reference trajectory used is a long looping motion occurring over $t_{f}=120$ s. Providing this reference trajectory to TumbleUCBound produces a W box constraint of $w_{max}=\left[0.147,0.0385,0.1236,0.0053,0.0049,0.0016\right]$ for the duration of the entire maneuver, the result of n=100 Monte Carlo propagations of inertia disturbances derived from approximate statistics of the visually estimated parameters. In practice for this sample study, a smaller $w_{max}$ was required for tightened constraint feasibility. This can be viewed as a feature of robust tube MPC—if constraints are tightened beyond feasibility, this is a sign that uncertainty levels must be improved and that robust control (given system constraints) is not enough to robustly counteract uncertainty. A smaller, less conservative stepwise bounded uncertainty is used in this sample analysis: $w_{max}=\left[0.05,0.05,0.05,0.00005,0.00005,0.00005\right]$. Note that reference trajectory velocity uncertainty is less impacted by Target uncertainty.

An ancillary gain matrix $K_{anc}$ was computed using an LQR formulation for Q=diag([1,1,1,10,10,10]) and R=diag([1000,1000,1000]), and the discrete dynamics of Eq. 4 with dt=0.2 s. An example of the constraint tightening procedure can be shown for $\bar{U}\in U$. Initial constraints are provided by an input box constraint U, with $u_{max}=0.4$ [N]. Other parameters are set as in Section 2.3. After computation of the robust positively invariant set (RPI), constraint tightening via the procedure in (Limon et al., 2008) is performed, resulting in a new set of polyhedral constraints:

$$A_{u}u=b_{u},$$ (28)

$$A_{u}=\left[\begin{array}{c} I_{3\times 3}\\ -I_{3\times 3} \end{array}\right],$$ (29)

$$b_{u}={\left[\begin{array}{cccccc} 0.239 & 0.239 & 0.239 & 0.239 & 0.239 & 0.239 \end{array}\right]}^{⊤}.$$ (30)

**FIGURE 9:**
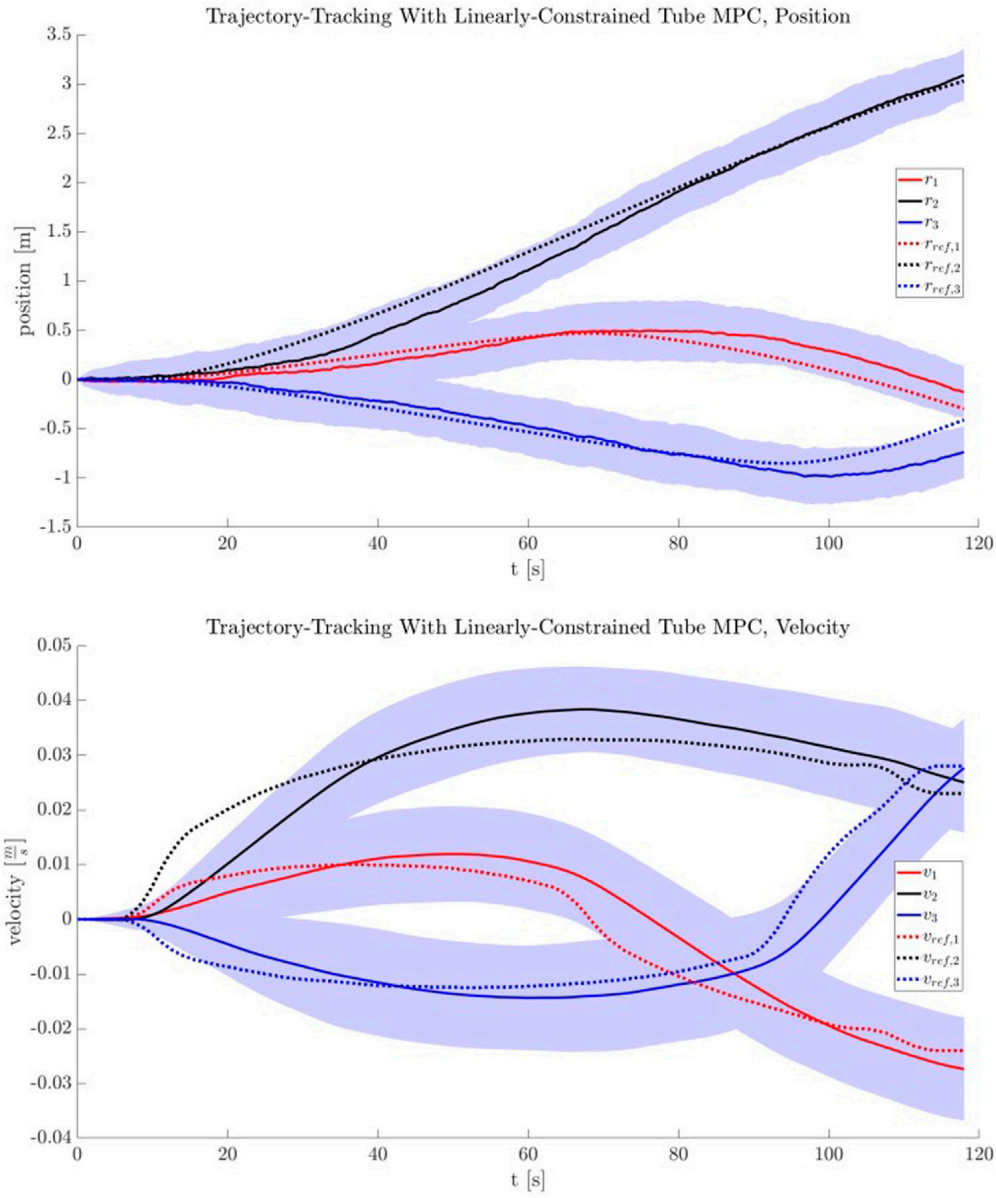
The position and velocity tracking performance for the robust tube MPC, with shading indicating 1-σ bounds of n=100 Monte Carlo trials. Note that nominal MPC and ancillary controller tuning can have a significant impact on controller performance (as with any MPC).

The tightened constraints are passed off to the robust tube MPC, which computes a nominal MPC solution at each time step, supplemented by the action of the ancillary controller. A series of n=100 Monte Carlo trials using this reference trajectory and uncertainty bound were performed to evaluate the tube MPC’s performance. To approximate w, a uniform distribution of $w∼U\left(-w_{max},w_{max}\right)$ was used to simulate the stepwise reference trajectory uncertainty. In reality, this trajectory uncertainty is caused by the differing Target tumble dynamics coupled with online trajectory updates (and is shown in Section 4.4). The averaged tracking results of this procedure are shown in Figure 9 for position and velocity tracking. The robust tube MPC was implemented using the CasADi optimal control framework (Andersson et al., 2019). Runtimes for a CasADi-based implementation with N=10, dt=0.2 were $\mu_{t}=0.014$ s, $\sigma_{t}^{2}=1.89\times 10^{-5}$ [s^2^] per step on a standard quad core Intel Core i7-4700MQ desktop system.

### 4.4 The Full Pipeline: A Case Study Result

A case study is presented for a full pipeline run of the proposed rendezvous algorithm for a scenario representing planned testing on the ISS using NASA’s Astrobee robotic satellites. To provide additional context on the computing environment, the Astrobee Robot Software uses the Robotic Operating System (ROS) as middleware for communication, with approximately 50 nodelets running on two ARM processors (MLP and LLP). These processors run the general-purpose operating system Ubuntu 16.04 and ROS Kinetic with a sensor suite including time of flight cameras, IMUs, and more as previously detailed.

Simulation results for this scenario were obtained using NASA’s ROS/Gazebo-based Astrobee simulation environment. The simulation environment includes extensive modeling of Astrobee including its impeller propulsion system, onboard visual navigation, environmental disturbances, and many more true-to-life models Flückiger et al. (2018). The full rendezvous pipeline was implemented as an additional set of Python and C++ ROS nodes and nodelets that run alongside the default Astrobee flight software. Significant development effort was dedicated to architecting software that would be usable for both simulation and hardware testing Albee et al. (2020). It may be helpful to the reader to follow Figure 4, which fully delineates the flow of information in the rendezvous pipeline.

#### 4.4.1 The Scenario

For this scenario it is assumed that the participant Astrobee robots have been placed at their respective starting positions within the Japanese Experiment Module (JEM) and are initially station-keeping. The relative distance between the two robots is 1.5  [m] in the y-axis of the ISS world frame, W. Both robots begin at rest, with the Target facing forward (positive x-direction) and the Chaser facing port (negative y-direction).

The Target performs a tri-axial tumble mimicking Envisat’s inertia $I_{ES}$ with initial angular velocity $\omega_{T}\left(0\right)=\left[0,3.53,3.53\right]$[deg/s] and maintains its translational position in the ISS inertial frame. The Chaser conducts Target observation at its initial position and maintains this position while the Target state and principal axes are estimated, the motion plan for a rendezvous maneuver with a duration of 60 s is generated, and the controller parameters determined. When the starting time for the approach provided by the motion planner is reached, the Chaser begins its approach to the MP.

#### 4.4.2 State and Principal Axes Estimation

**FIGURE 10:**
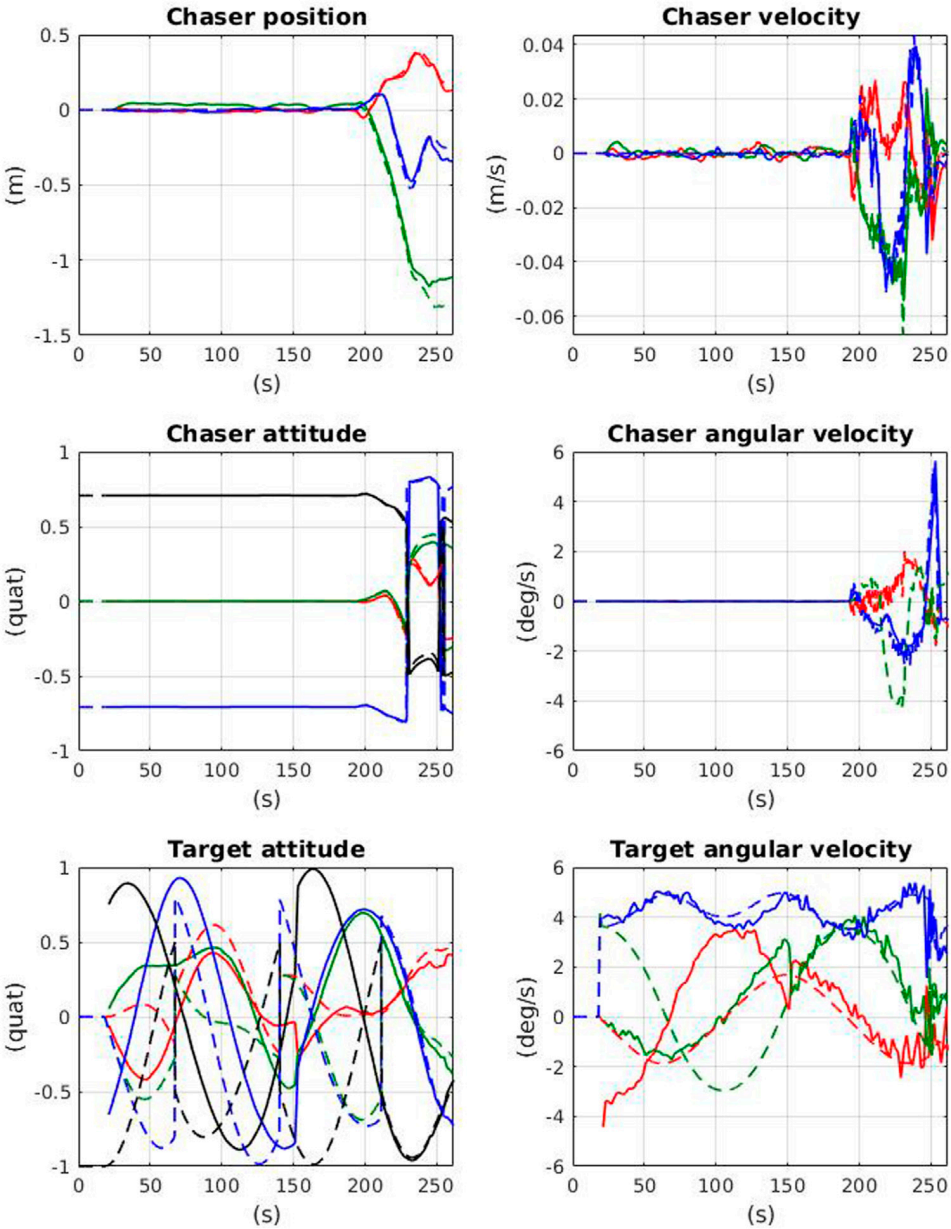
Chaser and Target state estimates over the time-history of the full pipeline demonstration. Solid lines indicate estimated values, dashed lines indicate simulator truth. Notice the “snap” that occurs in the Target estimates when the principal axes are determined (∼150 s).

The Chaser successfully observed the Target’s tumbling motion and provided accurate state estimates for both spacecraft throughout the entire maneuver. The observation period lasted 120 s to accumulate enough Target angular velocity estimates before determining the orientation of the principal axes. Figure 10 shows the time history of state estimates throughout the maneuver. The “snap” in Target’s attitude and angular velocity estimates is clearly evident when the principal axes are determined and proper alignment occurs. The average time taken for a factor graph update was 0.08 s on the same machine as Section 3.5. These values were then handed off to the Chaser motion planner.

#### 4.4.3 Chaser Motion Planning

**FIGURE 11:**
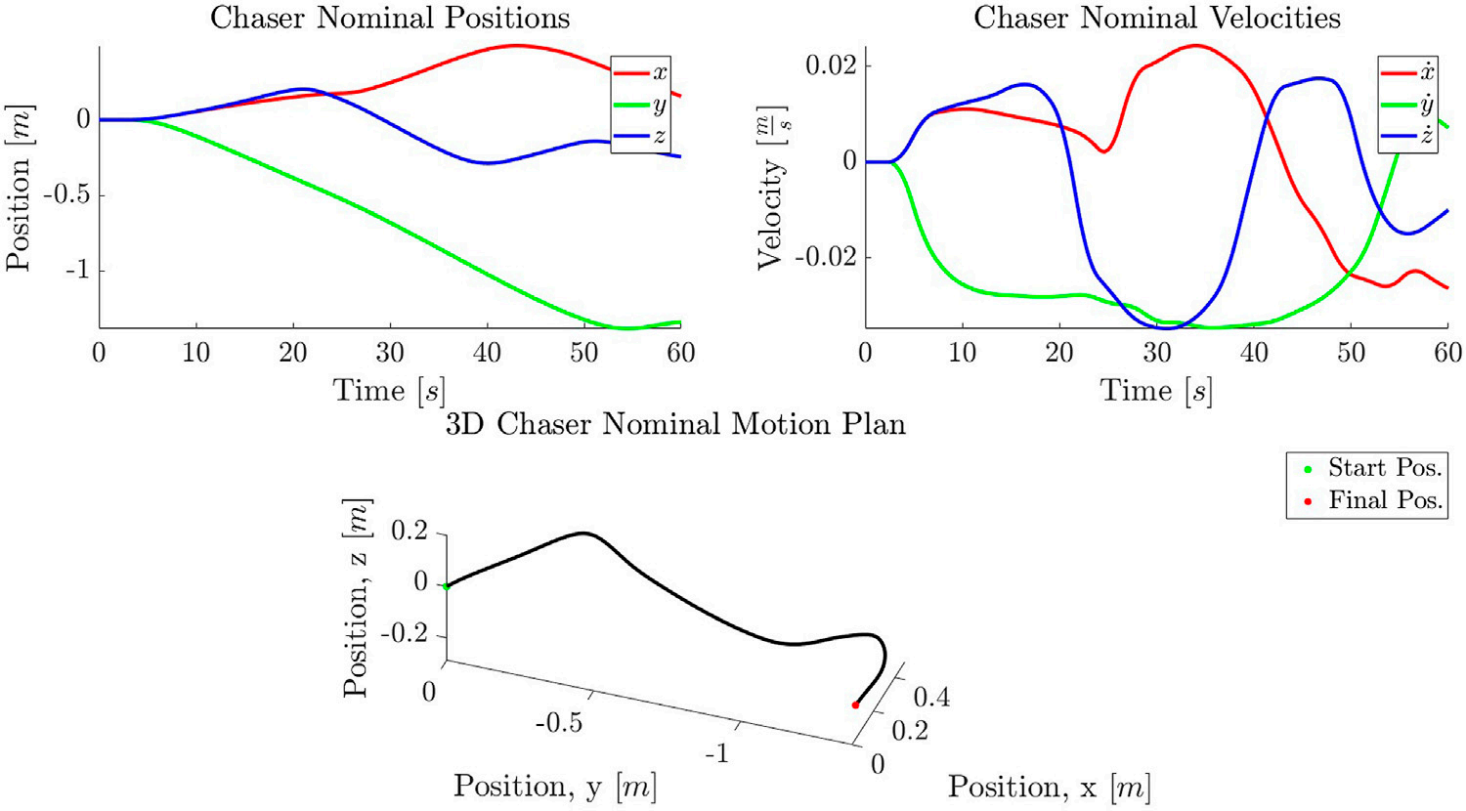
A planned trajectory in position **(A)** and velocity **(B)** for a tri-axial tumble query with inputs supplied by the Target estimation, with final Chaser position and velocity equal to that of the MP. The 3D representation of the trajectory is given **(C)**. The trajectory is presented in the inertial frame.

The estimated Target parameters and the measured Chaser and estimated Target positions in the inertial frame are of interest for the motion planner. The motion planner uses the LUT designed for a given set of Target parameters as indicated in Section 3.3 to determine the warm-start parameters for the given Target parameter query and from which a motion plan is developed. Included in this plan are Chaser states at each via point and a start time for the approach maneuver. A planned trajectory for this scenario is provided in Figure 11, using the outputs of the visual estimation procedure.

For the tumble described in this case study, the motion planner produces plans with 100% success for random initial conditions. The mean time for plan generation for this scenario is 1.12 s and the 3σ is 3.12 s.

#### 4.4.4 Uncertainty Bound and Robust Tube MPC

The resulting motion plan, xrefB(t0:N) (defined in the body frame) and $u_{ref}\left(t_{0:N}\right)$, is handed off to the robust controller as in Figure 4. The primary ingredients remaining before robust tube MPC can begin tracking are an approximation of the robust invariant set (RPI), ℤ and accompanying tightened input and state constraints, and real-time updates of the estimated Target orientation ${\hat{q}}_{T}\left(i\right)$.

The first step in calculating these values is to determine the uncertainty bound, w. The motion plan is handed off to the uncertainty bound approximation module along with the vital full statistics of estimated values from the visual estimation: $N\left({\hat{q}}_{T}\left(0\right),\Sigma_{q}\right)$, $N\left({\hat{\omega }}_{T}\left(0\right),\Sigma_{\omega }\right)$. Using the algorithm of Figure 6, the uncertainty bound can be approximated. In this case, the stepwise uncertainty due to Target estimation inaccuracy is w=[0.0145,0.0065,0.0152,0.0024,0.0012,0.0027], representing stepwise error in reference trajectory positions and velocities.

Now, w is handed off to the robust set approximation method detailed in Section 3.5 along with other vital information including the disturbance rejection gain $K_{anc}$, system dynamics f(x,u), and constraints as detailed in Figure 4. The goal is to produce the robust set approximation ℤ, with feasible state and input constraints. In this particular scenario, the uncertainty levels are too great for the available thruster authority from U; this is a useful feature, indicating that an absolute robustness guarantee for this particular level of uncertainty is not possible, and prompting two options. The agent could, in theory, choose to reobserve and replan for new Target statistics, thus increasing confidence in the Target motion. On the other hand, it is possible to obtain a safety guarantee for a reduced uncertainty bound, $w_{s}$ for a less conservative *σ* of the uncertainty approximation. This can still provide a robustness certification for a likely level of uncertainty.

**FIGURE 12:**
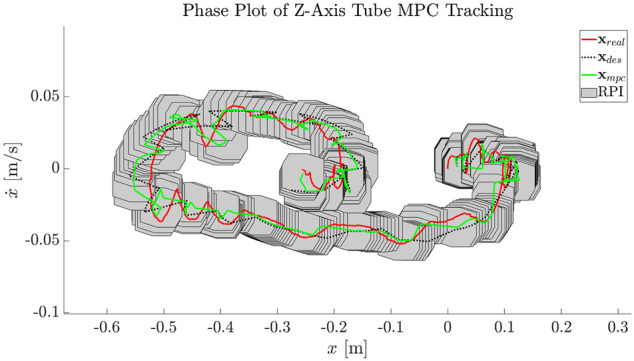
An example of the robust tube MPC maintaining the real x (red) within an invariant set (gray polytope) centered on the nominally planned MPC solution (green). Here, the RPI is shown for the first timestep of each receding horizon MPC solution.

**FIGURE 13:**
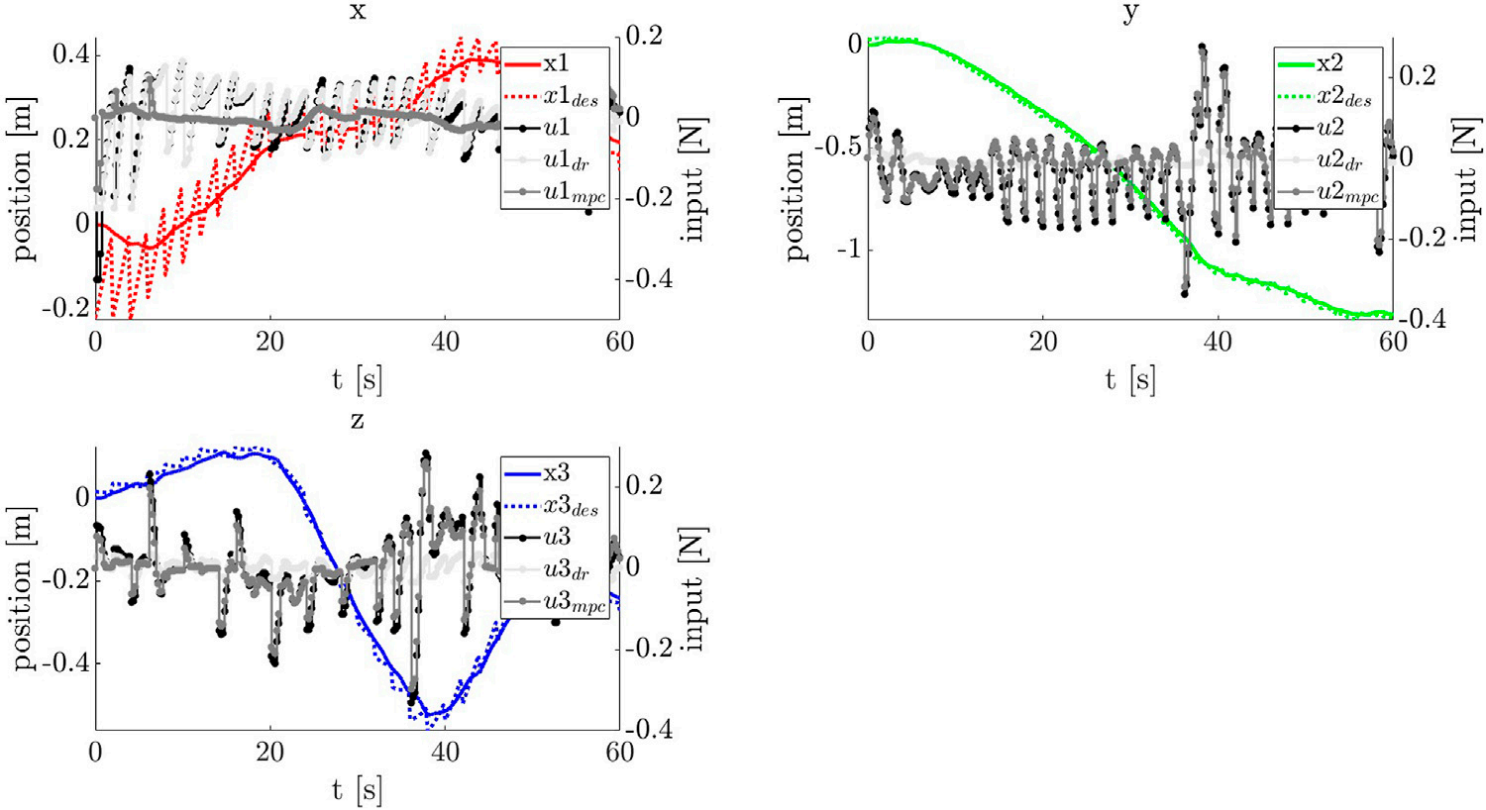
The input and state histories for the robust controller tracking the *online-updated* reference trajectory (dashed). It is important to remember that the reference trajectory in this scheme actually shifts in real-time as new SLAM estimates of the true Target attitude, ${\hat{q}}_{T}$ become available. It is precisely this stepwise shifting for which the tube robustness guarantee is produced. $u_{dr}$ indicates the disturbance rejection component, while $u_{mpc}$ indicates the nominal MPC component.

**FIGURE 14:**
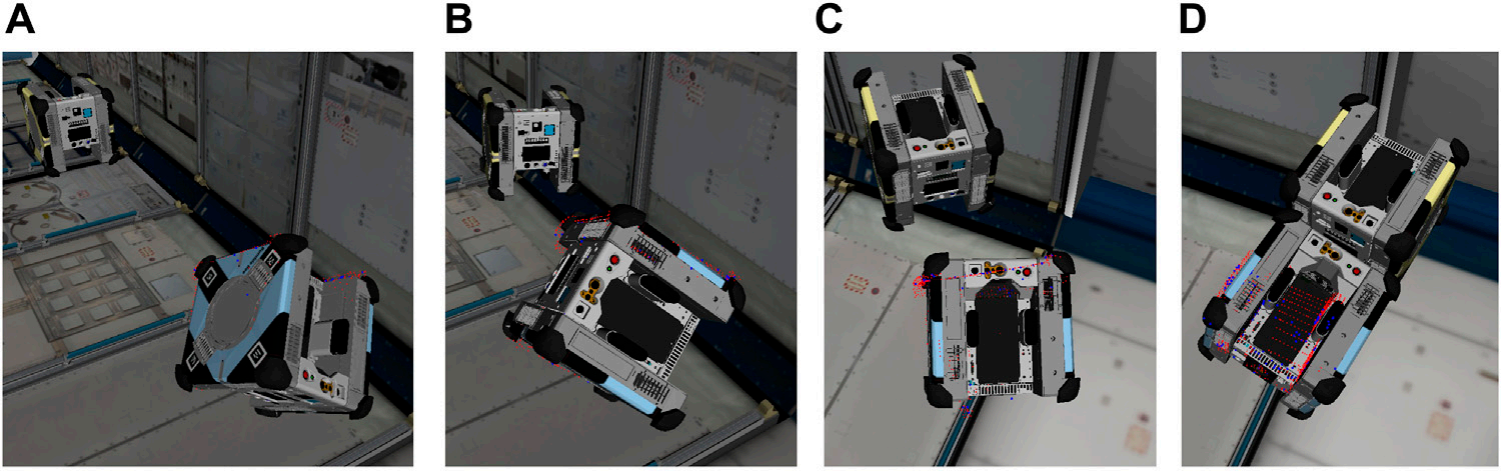
A timelapse of the rendezvous portion of the pipeline for an arbitrary tri-axial tumble. **(A–D)**, the Chaser begins tracking the motion plan with its robust controller while periodically updating the inertial frame reference trajectory based on SLAM estimates of ${\hat{q}}_{T}$.

In this case, a shrunken $w_{s}$ is used resulting in an approximation of the RPI visible in Figure 12, along with tightened input and state constraints. Now, the robust tube MPC is operated, constantly adjusting xrefB(t0:N) to inertial frame coordinates based on an updated ${\hat{q}}_{T}\left(i\right)$. The resulting track, showing the online-updated reference trajectory in dashed lines, is shown in Figure 13. The shifting of the reference trajectory is accounted for in the robust tube MPC setup; this is visible in Figure 12, where the RPI centered on the nominal MPC plan always contains the real trajectory. The MPC runs at 5 Hz over a 5 s horizon with a computation time of N(7.2,1.6)  ms on the machine used in Section 3.5. Even assuming a generous ten-fold computational slowdown on the Astrobee processors (a value that has held in practice) this timing is well within the required 200 ms desired. The result is a successful track to the MP, as shown in the simulation progression of Figure 14.

## 5 Conclusion

The framework and algorithms proposed in this study are a significant step toward autonomous rendezvous with tumbling Targets, uniting multiple key algorithmic components of the autonomy pipeline. Furthermore, key uncertainty sources are considered throughout and robustness and constraint satisfaction despite these uncertainties are incorporated into the pipeline logic. The treatment of uncertainty due to imperfect Target estimation combined with online-updating is considered, along with the implications of choosing overly conservative uncertainty bounds. Planned ISS tests in mid-2021 on the Astrobee platform will provide extensive experimental validation of this study, which has been shown algorithmically defined here and demonstrated in a detailed simulation environment that directly transfers to the Astrobee hardware.

Some major lessons learned from the development of this framework include the need for early standardization and the practical difficulties of moving to hardware implementation. Hardware implementation is vital, giving a direct look at the actual sensors, noise, computational power, and environments that will be seen by the algorithms developed for autonomous systems. However, hardware implementation leads to many complications, particularly in moving from desktop-based computational tools to embedded programming that may be lacking important libraries or computational power for example. Additionally, because of the number of algorithmic components, standardization and message-passing procedures must be settled early in development; luckily, ROS takes care of some of this complexity on the Astrobee platform. Some practical lessons learned are further documented in (Albee et al., 2020).

Algorithmically, the estimation, motion planning, uncertainty propagation, and robust control components have been outlined in detail and their application to a relevant satellite system explained. Individual performance metrics have been analyzed, showing the effectiveness of each of these portions independently. Additionally, the full pipeline presented in Figure 4 has been shown, demonstrating success of the autonomous rendezvous procedure in a detailed simulation environment, in real-time. A significant next step is ISS demonstration of the proposed pipeline on Astrobee hardware.

An interesting future direction for this study from a motion planning and controls perspective is the determination of system unknowns during execution. Prior work in this area for robotic free-flyers has recently been proposed and is applicable here in the case of online motion planning that accounts for the learning of Target inertial properties and permitting online recomputation, for instance (Albee et al., 2019). As it stands, the robust rendezvous framework documented here creates a full framework of algorithms needed to perform autonomous rendezvous with uncertain tumbling targets and adds robustness and systems integration considerations to an important open problem in microgravity close proximity operations, and demonstrates its effectiveness in a detailed simulation environment. Future study will document the framework’s performance as it moves toward on-orbit hardware demonstration.

## Data Availability

The raw data supporting the conclusion of this article will be made available by the authors, without undue reservation.

## Footnotes

1 The polhode is the path traced by the angular velocity vector of a rotating body on its inertia ellipsoid.
